# Emergence of multidrug-resistant blaCTX-M-65/gyrA_D87Y clones among the circulating Salmonella Infantis population in Mexico

**DOI:** 10.1099/mgen.0.001645

**Published:** 2026-02-16

**Authors:** Enrique Jesús Delgado-Suarez, Aliannys Lázara Puente-Cruz, Luisa María Sánchez-Zamorano, María Salud Rubio-Lozano, Nayarit Emérita Ballesteros-Nova, Cindy Fabiola Hernández-Pérez, Montserrat Hernández-Iturriaga, Elisa Cabrera-Díaz

**Affiliations:** 1 Facultad de Medicina Veterinaria y Zootecnia, Universidad Nacional Autónoma de México, Ciudad de México, México; 2 Instituto Nacional de Salud Pública, Dirección de Enfermedades Crónicas, Centro de Investigación en Salud Poblacional, Cuernavaca, Mexico; 3 Centro Nacional de Referencia de Inocuidad y Bioseguridad Agroalimentaria, Estado de México, Mexico; 4 Departamento de Investigación y Posgrado de Alimentos, Facultad de Química, Universidad Autónoma de Querétaro, Querétaro, Mexico; 5 Departamento de Salud Pública, Centro Universitario de Ciencias Biológicas y Agropecuarias, Universidad de Guadalajara, Guadalajara, Mexico

**Keywords:** chickens, genomic epidemiology, multidrug resistance, *Salmonella* Infantis

## Abstract

Global dissemination of emergent multidrug-resistant (MDR) *Salmonella* Infantis (ESI) is of great public health concern. ESI exhibits increased virulence and MDR phenotypes, features conferred by a conjugative megaplasmid (pESI-like). In Mexico, the potential circulation of ESI clones has been overlooked. This study assessed the structure, diversity, genomic features and transmission dynamics of *Salmonella* Infantis isolates (*n*=191) from cattle, pigs, chickens, humans, surface waters and the environment from surveys conducted by research laboratories and government agencies in the 2008–2024 timeframe. Three genomic approaches were implemented: blast analysis, SNP-based phylogenetic analysis and ancestral state reconstruction analysis. The phylogeny divided the population into two divergent sublineages, based on the presence/absence of pESI-like plasmids. The apparently recent and massive acquisition of these plasmids had a profound impact at the population level, changing the proportion of isolates with MDR genotypes from 0 to 68%. Although ESI was mostly associated with chickens, it was also present in surface waters. In fact, our ancestral state reconstruction analysis showed that ESI likely evolved from surface water ancestors within poultry production environments. Most locally circulating ESI clones (73%) harbour resistance factors to 6–8 antibiotic classes, including extended-spectrum cephalosporins (*bla_CTX-M-65_*) and fluoroquinolones (*gyrA* D87Y mutation), a feature shared with global ESI variants that we coined as *CTX-M-gyrA*6-8. Control measures are urgently needed to prevent further dissemination of ESI, as well as the systematic surveillance of *CTX-M-gyrA*6-8 clones, particularly in poultry, to reduce the risk of human exposure to this potentially deadly pathogen.

**Impact Statement**

The global emergence of multidrug-resistant (MDR) *Salmonella* Infantis (ESI) has profound implications for public health. Invasive infections caused by ESI may have fatal outcomes because several MDR clones can grow in the presence of critically important antibiotics. Acquisition of a conjugative pESI-like megaplasmid conferring increased virulence and multidrug resistance is considered a hallmark of rapid ESI expansion. This is apparently a breakthrough in the evolution of serovar Infantis, as it leads to an overrepresentation of ESI at the population level, coupled with a marked increase in the proportion of MDR strains. Currently, this process occurs mainly within poultry production environments, which appear to be the inception of ESI. Further recombination within pESI-like plasmids generates various ESI clones, some with intensified antimicrobial resistance, which are disseminated throughout the food chain and the environment.

## Data Summary

The raw sequences and genome assemblies used in this study are publicly available at the National Center for Biotechnology Information (https://www.ncbi.nlm.nih.gov/pathogens) under BioProjects PRJNA313928 and PRJNA480281. The individual accession numbers are provided in this article and as supplementary information (Table S1). Supplementary information on this study is available at the Figshare portal through the following link: https://doi.org/10.6084/m9.figshare.30247702 [1]

## Introduction

Currently, a worrisome proportion of non-typhoidal *Salmonella enterica* strains have acquired resistance to existing treatments. Therefore, this bacterium was included in the high priority group of the World Health Organization [2]. In this regard, the globally emergent multidrug-resistant (MDR) *S. enterica* subsp. *enterica* serovar Infantis (ESI), which is mostly found in poultry and ill patients, poses a critical public health risk [3].

In-depth genomic analyses revealed that ESI harbours a megaplasmid (pESI) carrying integrons and antimicrobial resistance (AMR) genes to aminoglycosides, sulphonamides and tetracyclines [4]. Moreover, some ESI clones carry *bla_CTX-M_* genes and *gyrA* point mutations, which encode additional resistance to critically important extended-spectrum cephalosporins and fluoroquinolones, respectively [3,5]. Furthermore, these strains have a particular set of virulence factors that are associated with increased virulent phenotypes [5] and are believed to contribute to their successful dissemination [4]. This pattern includes K88-like fimbrial clusters, toxin/antitoxin systems and operons encoding a yersiniabactin siderophore and mercury resistance, among others [3,4].

The virulent and MDR traits of ESI have serious implications for public health, particularly in low- and middle-income countries (LMIC), where resources are limited for treating infections caused by these strains in clinical settings. Moreover, the intense trade of foods in the global market further highlights the epidemiological significance of ESI because of its increased ability to disseminate [5,6]. These considerations underscore the need for further monitoring of ESI in LMIC, where most research is limited to the prevalence of *Salmonella*, serovar diversity and AMR phenotypes [7,8].

As of 4 February 2025, *Salmonella* Infantis accounted for nearly 40% (182/493) of the poultry isolates from Mexico uploaded to the National Center for Biotechnology Information (NCBI) Pathogen Detection database [9]. Recently, this serovar was linked to human infection [8]. However, no evidence supports the circulation of ESI clones.

*Salmonella* isolates recovered from chickens in Mexico have been analysed based on virulence genes [10] and multilocus sequence typing [11]. Broader genomic approaches have been used to assess genetic relatedness and strain typing based on virulence factors, AMR genes and plasmids in chicken-associated *Salmonella* serovars [12]. Nonetheless, little is known about the evolutionary dynamics within the serovar Infantis population, including data on ESI prevalence and genomic features. To fill this knowledge gap, this study assessed the structure of the *Salmonella* Infantis population circulating in Mexico. For this purpose, we identified ESI strains based on their typical genomic features (pESI-like plasmids, virulence and AMR genes) and conducted phylogenetic analysis and a preliminary assessment of the pathogen ecology and transmission dynamics using ancestral state reconstruction analysis.

## Methods

### The *Salmonella* Infantis genomes dataset

**Fig. 1.**
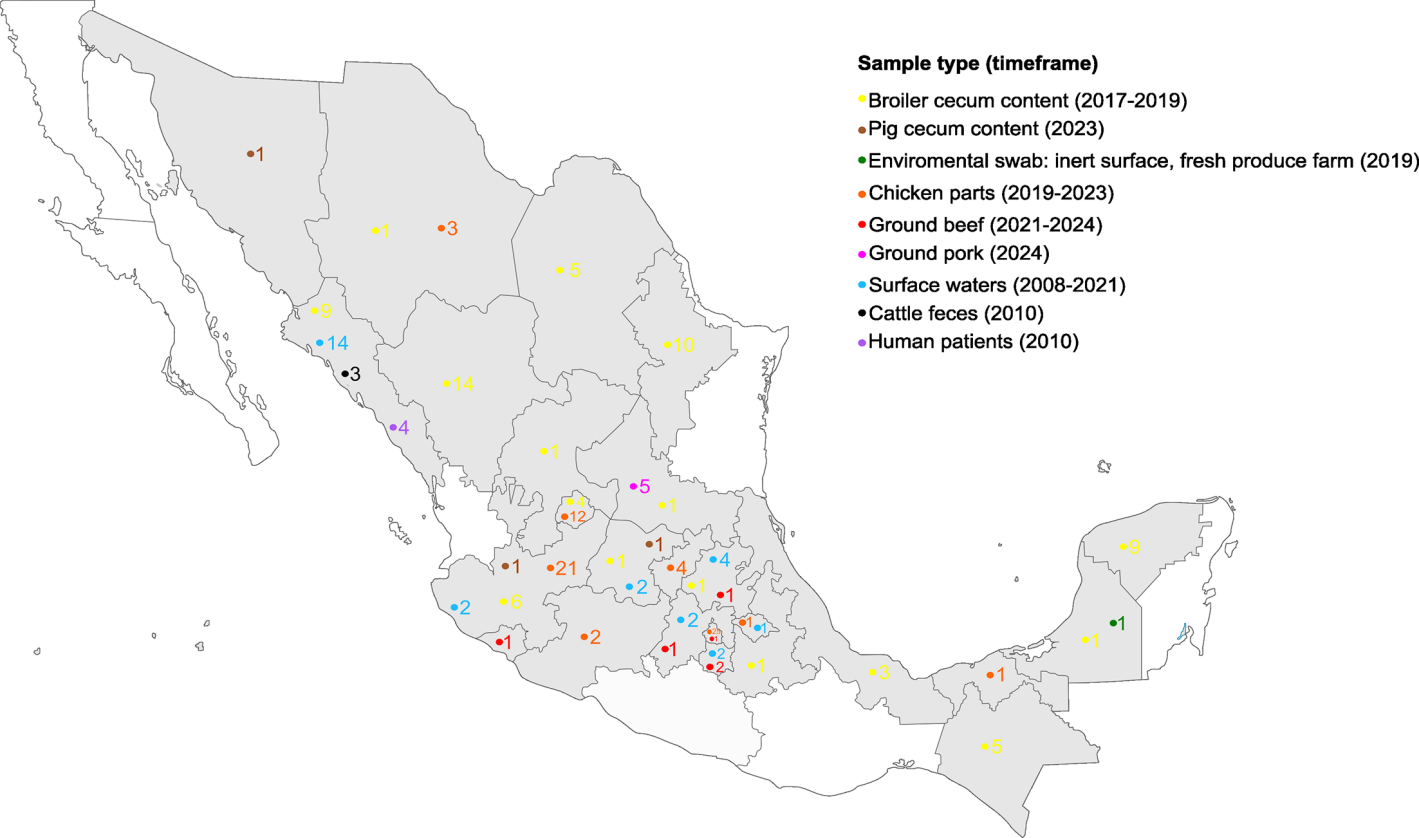
Number of *Salmonella* Infantis isolates collected in 27 Mexican states (regions in grey) according to sample type and timeframe.

In this study, we analysed a dataset of 191 *Salmonella* Infantis strains isolated from various sources throughout Mexico (Fig. 1). Most of these isolates (*n*=171) were collected during the 2017–2024 period. Strains from broiler and pig cecum contents and an environmental swab were collected by the competent body of the Mexican Ministry of Agriculture as part of official surveillance programmes at broiler (2017–2019 timeframe), pig (2023) and fresh produce farms (2019). Likewise, the competent body of the Mexican Ministry of Health collected 12 isolates during official surveillance of raw chicken at retail stores in 2023.

Additional strains were collected by three Mexican universities (Faculty of Chemistry, Autonomous University of Queretaro; Department of Public Health, University of Guadalajara; and Faculty of Veterinary Medicine, National Autonomous University of Mexico) and the National Institute for Public Health. Laboratories from these institutions conducted recent surveys of retail stores and isolated strains from raw chicken parts (2019–2023), beef (2021–2024) and pork (2021–2024). Finally, another 14 strains were isolated from surface waters in the proximity of agricultural areas (2019–2023 timeframe) by laboratories from the Faculty of Veterinary Medicine, National Autonomous University of Mexico; the Public Health Department, University of Guadalajara; and the Food and Development Research Center of Sinaloa.

As historical strains, we also included 20 isolates collected by the National Laboratory for Research in Food Safety during the 2008–2010 period. Part of these strains (*n*=13) were isolated from surface waters (rivers and irrigation canals) in Culiacan, the capital of the Northern state of Sinaloa and one of the major agricultural regions of Mexico. The remaining strains were isolated from cattle faeces and human patients in 2010.

All genome assemblies were downloaded from the NCBI Pathogen Detection database (https://www.ncbi.nlm.nih.gov/pathogens) through the accession numbers provided in Table S1, available in the online Supplementary Material. For isolates lacking assemblies (*n*=143), we downloaded the raw reads and conducted a *de novo* assembly using SPAdes version 3.1.3.1 (RRID:SCR_000131) [13]. The assemblies were subjected to serovar prediction using SISTR version 1.1.1 (RRID:SCR_024342) [14], and the indicated serovar of each genome was confirmed to be correct.

Overall, the strains analysed in this study were isolated from eight different sources (broiler and pig cecum contents, raw chicken parts, beef, pork, surface waters, cattle faeces and human patients) in 27 of the 32 Mexican states over 17 years (2008–2024). For comparative analysis, strains from broiler cecum contents and chicken parts were classified as ‘chicken isolates’, those from pig cecum contents and ground pork as ‘pig isolates’ and those from cattle faeces and ground beef as ‘cattle isolates’. As of 4 February 2025, these isolates represented 100% of all the *Salmonella* Infantis genomes sequenced in Mexico that are publicly available in the NCBI Pathogen Detection database (https://www.ncbi.nlm.nih.gov/pathogens).

### Identification of ESI strains

ESI strains were identified based on the presence of pESI-like plasmids in the assemblies. For this purpose, we performed a local blast analysis of the pESI reference sequence (NCBI accession CP047882.1) against our genome assemblies (in fasta format) using blast+ version 2.10.1 (RRID:SCR_004870). To reduce the probability of obtaining random hits, we ran a blastn analysis with an e-value cutoff=10^−30^, and positive hits were required to have a minimum query coverage of 90%.

The isolates were predicted to carry pESI-like plasmids and thus considered ESI strains if the draft genomes had positive hits for the key pESI virulence genes (K88-like fimbrial clusters, toxin/antitoxin systems, a mercury resistance operon and the yersiniabactin siderophore) [15] and ≥70% of the reference pESI coding sequences, which may include class-1 and class-2 integrons and their corresponding AMR gene cassettes (*aadA1*/*qacEDelta1*/*sul1* and *dfrA14*, respectively), as well as other pESI AMR genes that are not associated with integrons: *aph(3′)-Ia*, *aph(4)-Ia*, *aac(3)-IVa* and *tet(A*) [3,4]. In addition, AMRFinderPlus version 4.0.19 was used to determine the AMR genotypes (both genes and point mutations) of the isolates. The results of the blast analysis and the AMR genotypes were used to generate a presence/absence heatmap of the genomic features and AMR genotypic profiles of the strains under study. To further corroborate the presence of the plasmid in the genomes, the pESI reference sequence was aligned to our study genomes in the Proksee web server [16].

### Genetic diversity of *Salmonella* Infantis at the population level

According to the previously described blast analysis, the isolates were grouped into nine genomic profiles based on the arrangement of integrons and the occurrence of MDR genotypes. Therefore, the population structure was assessed using representative genomes from each of the identified genomic profiles. We selected strains from all geographical regions, collection dates and AMR genotypes (i.e. pansusceptible, MDR) represented in that group, except for isolates from different locations with identical AMR genotypes. Using these criteria, 84 genomes were selected for analysis.

The assemblies were then subjected to SNP-based phylogenetic analysis. We used CSI Phylogeny version 1.4 [17] to locate, filter and validate the SNPs for the 84 genomes, with the following default values: 10× minimum depth at SNP positions, 10% minimum relative depth at SNP positions, 10 bp minimum distance between SNPs (prune), 30 minimum SNP quality, 25 minimum read mapping quality and 1.96 minimum *Z*-score. The closed genome of the *Salmonella* Infantis strain R21.1575 (accession CP121068.1) was used as a reference to obtain the multiple genome alignment and SNP matrix. Table S2 presents the SNP matrix.

The resulting alignment (1,384 SNP sites) was analysed by the maximum-likelihood (ML) method using IQ-Tree, version 3.01 (RRID:SCR_017254) [18] configured to search for the best-fit model (-m TEST option, which identified K3P+ASC+G4 as the best-fit model according to Bayesian information criterion) [19] and 1,000 bootstraps. The resulting tree was edited using iTOL version 7.0 (RRID:SCR_018174) [20], and the results of the previously described blast analysis were mapped onto the tree.

### Analysis of transmission dynamics and ancestral isolation source reconstruction

Initially, we planned to conduct a molecular clock analysis using the previously described phylogenetic tree to estimate the time of emergence of ESI within Mexico. Therefore, this tree was analysed using TempEst version 1.5.1 (RRID:SCR_017304) [21] to test for the presence of a temporal signal in the dataset. However, the regression model between the genetic divergence through time and sampling dates had a negative slope (−0.4), indicating that this dataset contains little or no temporal signal. The model residuals also had considerable variation, indicating that the specified date of sampling of most isolates did not correspond to the observed genetic distance (Fig. S1). Perhaps some isolates in the public database may have been labelled with incorrect sampling dates. Moreover, except for a few exceptions, these isolates were sequenced 2–9 years after collection. Therefore, excessive passaging may have also occurred during this period, leading to the observed increased genetic distance variability. For these reasons, conducting a molecular clock analysis was not feasible. As an alternative, we conducted a preliminary assessment of ESI transmission dynamics. Our dataset was appropriate for this purpose because the phylogeny included isolates from multiple sources and geographical regions within Mexico.

As a first step, we constructed a transmission network using StrainHub, version 1.1.2 [22]. For this purpose, the above-referred phylogenetic tree was used as input, along with a csv file containing isolate metadata (i.e. isolation source). Next, the software built a network by mapping the metadata onto the tree and performing a parsimony ancestry reconstruction step to create links between the associated metadata and generate the network transmission metrics: source/hub ratio and the centrality metrics betweenness, closeness and degree. Among these metrics, we were particularly interested in the source/hub ratio, which measures how important each node is as a source, hub or sink for the pathogen (source=~1, hub=0.5, sink=~0). In the transmission network, the arrows show the directionality of transition between states, whereas the thickness of lines and arrows represents the frequency of transitions (the thicker the more transitions) [22].

Transmission network analysis results were further corroborated using Mesquite software version 3.81 (RRID:SCR_017994) [23]. For this analysis, we added the isolation source to the terminal taxa as metadata in the phylogenetic tree. In this way, the isolation source is defined as a ‘character state’ and treated as a categorical variable that allows inferring the history of the character state evolution across the ancestral nodes of the tree. Hence, both the tree and character matrix of the metadata were subjected to the ‘trace character history’ analysis. We used both parsimony unordered and ML models (the ML under the Mk1 model: Markov k-state 1 parameter model). However, the tree had polytomies that needed to be solved before running the ML model. To address this issue, branch lengths ≤0 were arbitrarily assigned a length of 10^−6^. To facilitate reproducibility, the phylogenetic tree and character matrix are provided in File S1. The software installation instructions and documentation are available at https://www.mesquiteproject.org.

### Genetic relatedness among ESI clones circulating globally

Genetic relatedness between circulating ESI clones in Mexico and those reported globally was also investigated. For this purpose, a second phylogeny was constructed, following the aforementioned methodology, using 14 representative strains among the isolates from Mexico included in this study and 28 public isolates from across the world. To select these global isolates, we searched the NCBI Pathogen Detection database (as of 4 February 2025), filtering isolates by serovar (Infantis) and AMR genotypes (isolates carrying AMR genes to 6–8 antibiotic classes, including *bla_CTX-M-65_* and the *gyrA* D87Y mutation). From the resulting list, we selected one genome from each of the isolation sources represented within each country. Using this procedure, we selected 28 strains isolated from four different sources across 19 countries and 5 continents/regions. These strains were chosen because of their most worrisome AMR profile from a public health standpoint: resistance to 6–8 antibiotic classes, including extended-spectrum cephalosporins and fluoroquinolones [5,24]. The SNP matrix used in this analysis is presented in Table S3.

### Statistical analysis

The frequency distribution of isolates across the isolation sources (cattle, pigs, chicken, humans and surface waters/environment) was calculated. We also used Pearson’s Chi-square test to determine whether the occurrence of ESI strains was associated with the isolation source or the MDR genotype.

## Results

### Predominance of ESI at the population level

**Fig. 2.**
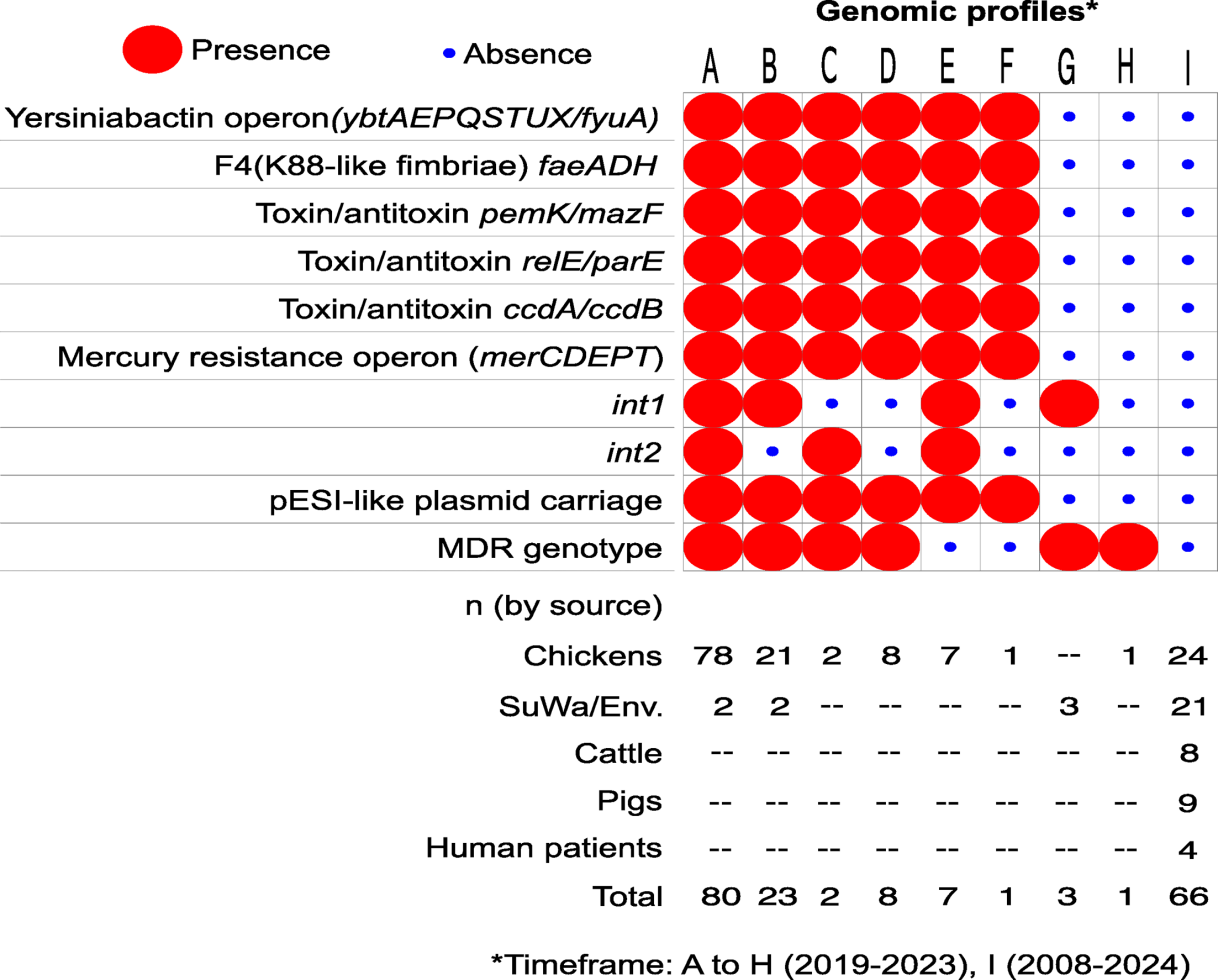
Frequency of pESI-like plasmid carriage along with pESI virulence genes, class-1 and class-2 integrons and MDR genotypes among 191 *Salmonella* Infantis strains isolated from various sources throughout Mexico. SuWa/Env.: surface waters and environmental samples.

blast analysis revealed that ESI strains are predominant in the *Salmonella* Infantis population under study. Over 60% of the isolates (121/191) carried the typical pESI-like virulence repertoire: the yersiniabactin siderophore operon, K88-like fimbrial clusters, mercury resistance operon and several toxin/antitoxin systems (Fig. 2). However, the distribution of integrons and MDR genotypes was uneven, leading to the identification of nine genomic profiles at the overall population level. The full report of the blast analysis results is provided in Table S4. The presence of pESI in the study genomes was also corroborated by the blast atlas analysis, showing the synteny with most of the pESI reference sequence (≥70%) in the isolates that were predicted to carry this plasmid (Fig. S2).

Chicken samples were the most significant source of ESI strains (*χ*^2^=117.0, *P*<0.0001), representing 96.7% (117/121) of the isolates carrying pESI-like plasmids. In contrast, no ESI strains were identified in pig, cattle or human isolates, whereas only 14% (4/28) were present in samples from surface waters (Fig. 2). Moreover, MDR genotypes were more frequently observed among ESI strains (*χ*^2^=143.6, *P*<0.0001), regardless of the isolation source. Furthermore, ESI predominance appeared to be recent (2019 onwards), as no ESI strains were identified among the historical isolates (2008–2010). Plasmid-borne virulence factors were not further considered in the comparative analysis, as these features were 100% conserved among the ESI strains.

### pESI-like plasmids: a milestone in the evolution of serovar Infantis

**Fig. 3.**
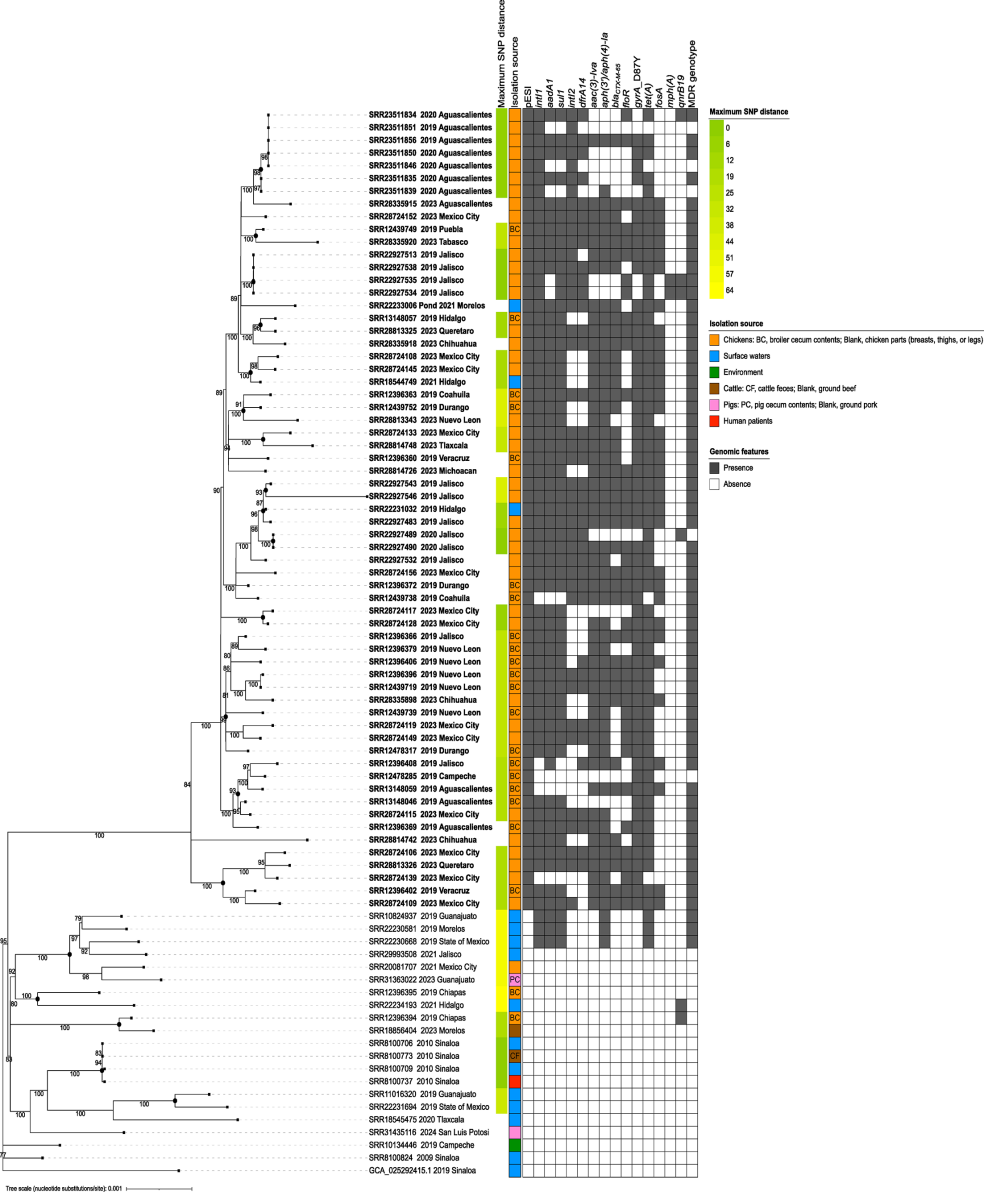
ML SNP-based phylogenetic analysis of 84 *Salmonella* Infantis isolates from Mexico (unrooted tree). Tip labels report the NCBI accession, year of collection and the Mexican state of origin. ESI strains are indicated using bold font. Statistical support (percent bootstrap) is indicated on the branches unless <70%. Maximum SNP distance within clusters (black circled nodes), isolation source and the occurrence of pESI-like plasmids, integrons and AMR genes are mapped onto the tree.

The SNP phylogeny broadly divided the strains into two divergent sublineages. Both sublineages significantly differed in terms of ESI profiles and AMR genotypes (Fig. 3). One sublineage comprised 21 isolates (from surface waters, the environment, cattle, pigs, chickens and humans), most lacking pESI-like plasmids and with a weak AMR profile, except for the 3 surface water isolates that carried MDR genotypes. Overall, this sublineage exhibited a higher genetic diversity than the one grouping all ESI strains, which formed multiple clonal clusters (0–20 SNPs) both within and across regions. The maximum genetic distance between any two ESI strains was ≤116 SNPs. At the population level, the genetic diversity was limited, with a maximum SNP distance of 161–224 among any two strains.

The heatmap next to the tree shows that pESI-like plasmids undergo substantial recombination, as indicated by the multiple arrangements of integrons and their associated AMR genes. This pattern was observed across isolation sources and geographical locations. The class-2 integron and its associated resistance cassette (*dfrA14*) were more frequently deleted in the ESI strains under study (Fig. 3). Interestingly, few chicken isolates (from Aguascalientes and Jalisco) carried both integrons but lacked most AMR genes and did not have MDR genotypes. Conversely, most of the counterparts from the same source and region had stronger AMR profiles.

Among other AMR genes commonly carried by pESI-like plasmids outside the integrons, the observed deletions involved more frequently phenicol (*floR*) and aminoglycoside [*aac(3)-IVa*, *aph(3′)-Ia*, *aph(4)-Ia*] resistance factors. In contrast, the acquisition of the *bla_CTX-M-65_* gene appeared to be a frequent outcome of the recombination process. This gene was widely disseminated among ESI strains from chickens and surface waters, which also had the chromosomal *gyrA* D87Y mutation, resulting in strong MDR genotypes. This AMR profile is more alarming because it confers resistance to 6–8 antibiotic classes, including critically important fluoroquinolones and extended-spectrum (third and fourth generation) cephalosporins. Therefore, we named this AMR genotype as ‘*CTX-M-gyrA*6-8’ to identify ESI clones with greater implications for public health. Among the ESI subpopulation, more than 70% of the isolates (88/121) had the *CTX-M-gyrA6-8* genotype (see Fig. S3).

### ESI likely evolved from surface water ancestors within poultry

**Fig. 4.**
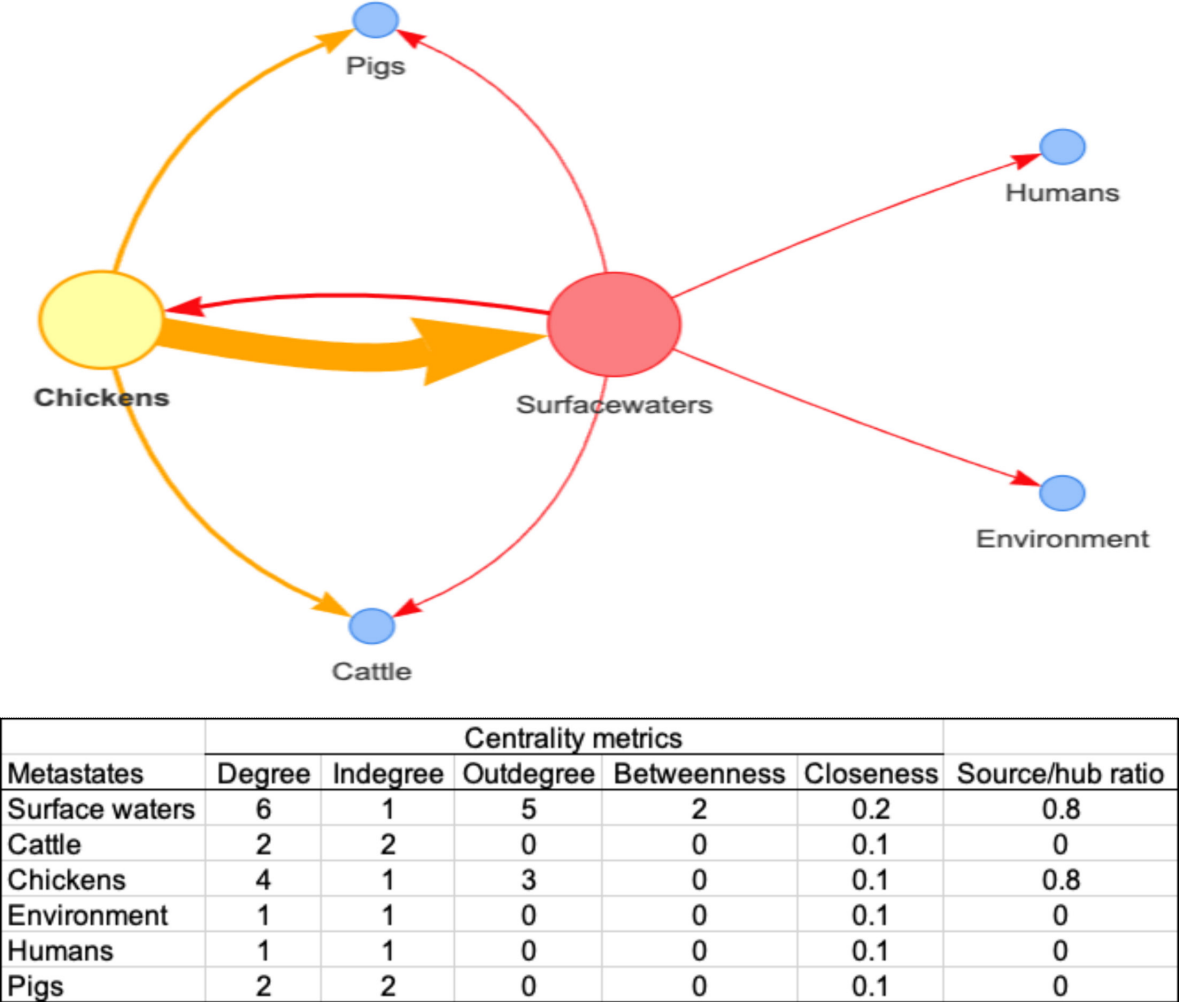
Transmission network of 84 *Salmonella* Infantis strains collected in Mexico from chickens (*n*=62), surface waters (*n*=15), cattle (*n*=3), pigs (*n*=2), environment and humans (*n*=1 each). The circles correspond to isolation sources, the arrows show the directionality and strength (arrow thickness) of transitions between sources and the metrics are reported under the network scheme.

According to the transmission network analysis (Fig. 4), both surface waters and chickens are the main reservoirs of *Salmonella* (source/hub=0.8). However, betweenness centrality indicated that surface waters were the most relevant hub for pathogen traffic, with transitions to every other source. Interestingly, only chickens were involved in transitions back to surface waters. Transitions originating from humans, pigs or cattle were not observed in this study. However, they cannot be discarded due to the limited availability of isolates from these sources in our dataset. As indicated by closeness metrics, which were >0 for every source, all of them may act as a direct transmission point to other nodes.

**Fig. 5.**
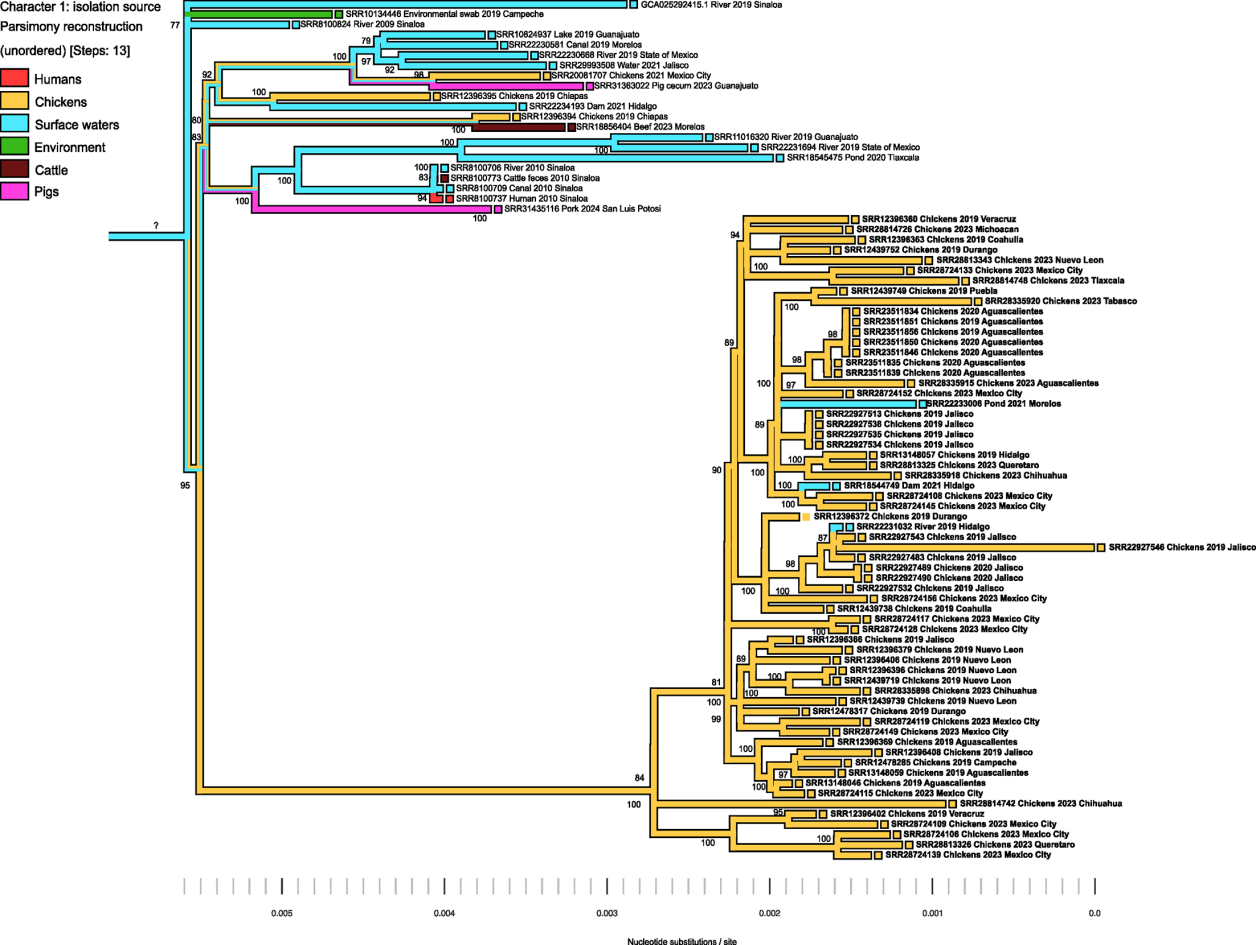
Isolation source history at ancestral nodes in a phylogenetic tree of 84 *Salmonella* Infantis strains. The reconstructed isolation source is colour-coded at each ancestral node. Statistical support (percent bootstrap) is indicated on the branches. The NCBI accessions, source, year of collection and location are indicated on the tip labels. ESI strains are indicated using bold font.

The reconstruction of the ancestral states further confirmed the central role of surface waters in pathogen transmission. The parsimony unordered model estimated that the most likely common ancestor of the study strains originated from surface waters. Similar results were obtained using the ML model, as surface waters had the highest proportional likelihood (0.99) of being the source of the common ancestor. The results of the parsimony model are presented in Fig. 5 because it is easier to interpret in graphical format, whereas those of the ML model are included in Fig. S4.

After each splitting event, the isolation source progressively changed from surface waters (at the most ancestral node) to a different source (or a mixture of all parsimonious sources). As proposed in the tree, although every isolate likely evolved from a surface water ancestor, they only evolved into ESI strains in chickens, which further disseminated to eventually reach surface waters again.

### Local *CTX-M-gyrA*6-8 ESI clone in the global context

**Fig. 6.**
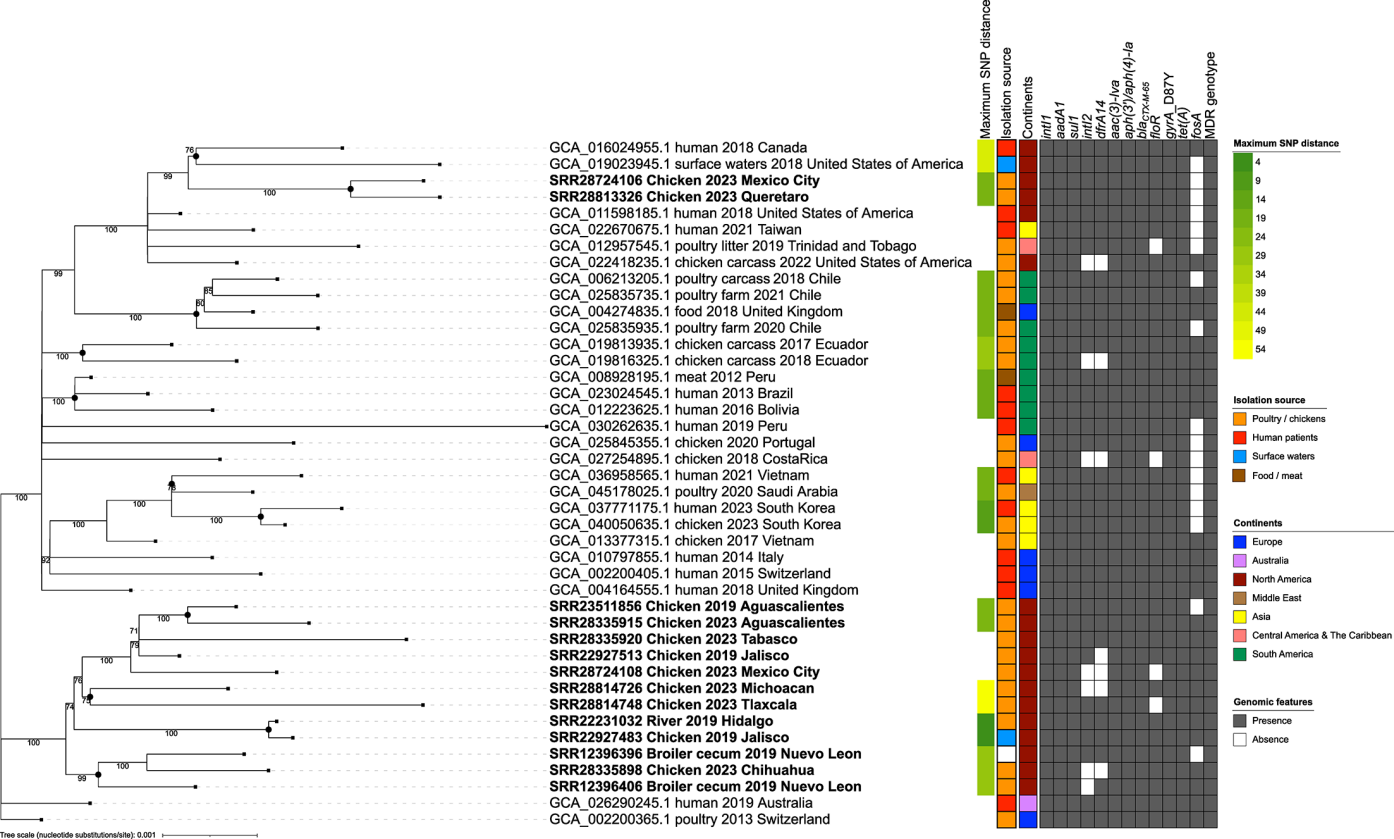
SNP-based phylogeny of 42 emergent *bla_CTX-M-65_/gyrA* D87Y *Salmonella* Infantis collected worldwide. Statistical support (percent bootstrap) is indicated on the branches unless <70%. Tip labels report NCBI accession, source, collection year and country of origin. Maximum SNP distance within clusters (black circled nodes), isolation sources, regions of the world and the AMR genomic features of the strains are mapped onto the tree. ESI strains from Mexico are indicated using bold font.

Phylogenetic analysis of MDR *bla_CTX-M-65_*/*gyrA* D87Y ESI strains collected worldwide showed limited genetic diversity (Fig. 6). Close genetic proximity (4–54 SNPs) was observed among strains collected from different sources, including human patients, both within and across countries, in several clusters (black circled nodes). The same occurred among strains from distant regions, such as Chile and the UK (14–20 SNPs), Vietnam and Saudi Arabia (19 SNPs) and Canada and the USA (46 SNPs). Notably, all possible pairs of the SNP matrix were within ≤112 SNPs, documenting both the local and global dissemination of this worrisome ESI clone.

Most ESI strains from Mexico formed a single subclade and were close (26–59 SNPs) to strains from Switzerland and Australia. The remaining two isolates from Mexico (SRR28813326 and SRR28724106, both from chickens) shared the same subclade with human, surface water and food-related strains from Canada, the USA, Taiwan and Trinidad and Tobago, which were 39–96 SNPs from them.

As shown in the heatmap next to the tree, AMR determinants were generally conserved among isolates from diverse sources and regions. Most of the observed variability involved the class-2 integron, which has a single resistance cassette (*dfrA14*). Thus, it makes a limited contribution to the AMR profiles of these strains. Moreover, few strains lacked phenicol (*floR*) and phosphomycin (*fosA*) resistance genes, but they still had MDR genotypes conferring resistance to 6–7 antibiotic classes.

## Discussion

Recent research from across the world revealed a rapid expansion of ESI exhibiting increased virulence and strong MDR genotypes [3,4, 6, 25, 26]. These features are sustained by the acquisition of a pESI-like megaplasmid harbouring multiple virulence and resistance factors [15]. The analyses conducted here identified a predominant lineage among the *Salmonella* Infantis population from Mexico, with the typical genomic profile of ESI [4]: presence of pESI-like plasmids harbouring the yersiniabactin operon, K88-like fimbrial clusters, several toxin/antitoxin systems, the mercury resistance operon, as well as class-1 and/or class-2 integrons and multiple AMR genes.

Apparently, this lineage diverged from the non-ESI subpopulation recently because no ESI strains were identified among historical isolates (2008–2010 timeframe). A similar trend was observed globally, whereby ESI was rarely reported before 2010 [3,4] but has been increasingly detected among recently sequenced serovar Infantis isolates worldwide [5,6, 24, 26]. In any case, this phenomenon had a profound impact at the population level, not only because of ESI predominance but also because of the dramatic increase in the proportion of isolates carrying MDR genotypes: it went from 0% in the non-ESI sublineage to >90% within the ESI subpopulation. These findings are consistent with recent reports showing that MDR *Salmonella* Infantis is among the most prevalent serovars circulating in chicken samples from Central Mexico [11,12].

Our results showed that chickens were the single most important source of this lineage, consistent with the results of multiple studies [3]. Moreover, among all the public *Salmonella* isolates collected from poultry samples in Mexico (*n*=493, according to the NCBI Pathogen Detection database as of 4 February 2025), those of serovar Infantis represented nearly 40% (182/493), quadruplicating the frequency of the next most abundant serovar (Enteritidis, 44/493). However, a few surface water isolates were also found to be ESI variants, consistent with the critical role of surface waters in *Salmonella* dissemination [27,28].

Although these findings are consistent with the reported association between ESI and poultry [3], our dataset contained a few isolates from other sources, including human patients, some of which were collected more than a decade ago. Hence, further research is needed to better understand the epidemiology of ESI in Mexico. Notwithstanding the complexity of this phenomenon, our results of transmission dynamics and reconstruction of ancestral isolation sources provided evidence on the risk of ESI dissemination in the environment.

Phylogenetic analysis confirmed that pESI-like plasmid acquisition is a hallmark of ESI evolution, leading to its divergence from the non-ESI subpopulation. Moreover, the pESI-borne genomic features appear to confer fitness advantages in chickens, given the recent emergence and strong epidemiological association of ESI with this species. In fact, our ancestral state reconstruction analysis strongly suggested that the acquisition of pESI-like plasmids and thus the evolution into an ESI strain occurred within poultry production environments. However, the factors governing this process are yet to be determined, paving the way for further and urgent research in this area.

In line with previous observations [5], we found evidence of recombination within pESI-like plasmids. Interestingly, as observed previously [4], this phenomenon involved integrons and AMR genes, but not virulence factors. Perhaps pESI-borne virulence genes are better indicators of the presence of ESI strains in draft genomes. The typing of pESI-like plasmids is cumbersome given their large size and complexity (they have arisen from the integration of several plasmids and multiple recombination events) [15]. Consequently, plasmid typing should be complemented with further in-depth genomic analyses to obtain more reliable results [26,29]. In this regard, a simpler approach could rely on confirming the presence of the specific virulence pattern of ESI [4], which is more stable than that of AMR genes. Such an approach could be implemented using cheaper techniques (i.e. PCR or microarrays), especially in settings with limited resources that cannot afford whole-genome sequencing for routine surveillance.

Our results also support previous observations of the circulation of ESI clones and genetically close ESI variants that appeared to have arisen from recombination within pESI-like plasmids [3,5]. In some cases, the outcome of recombination was the deletion of integrons and their associated resistance cassettes. The redundancy of certain AMR genes (especially for aminoglycosides and folate pathway inhibitors), contributed by both integrons and the chromosome, may result in higher fitness costs, leading to their eventual deletion, as observed in close to 23% of our study isolates and 17% of global strains. Although speculative, this reasoning is plausible because all ESI variants lacking integrons retained the MDR genotypes.

Unfortunately, plasmid recombination frequently resulted in the acquisition of the *bla_CTX-M-65_* gene, a phenomenon that was coupled with a chromosomal *gyrA* D87Y mutation, resulting in the alarmingly widespread AMR genotype that we coined *CTX-M-gyrA*6-8. This AMR genotype has been observed since 2013 in chicken isolates from Switzerland and more recently (2018–2023) across the world. This condition may lead to fatal outcomes in patients with invasive salmonellosis, which is usually treated with extended-spectrum cephalosporins or fluoroquinolones.

Comparative analysis using public isolates showed that these ESI variants also circulate worldwide, mainly in poultry and human patients, confirming the relevance of our findings from a public health perspective. Therefore, it is imperative to implement specific control measures to limit the dissemination of ESI. Moreover, to further reduce the risk of human exposure to this pathogen, systematic surveillance of *CTX-M-gyrA*6-8 *Salmonella* Infantis should also be considered, especially in poultry.

Previous research has revealed that the intestinal environment of several food-producing animals favours the conjugation of pESI-like plasmids, which could facilitate their dissemination to other *Salmonella* serovars and other enterobacteria [30]. This evidence supports the implications of our results regarding the need for systematic surveillance of *Salmonella* populations and the identification of factors leading to the acquisition of pESI-like plasmids. Both research areas should focus on poultry isolates, which, according to our results, fuel the growth of the ESI lineage.

In summary, this study documented the recent and somewhat overlooked emergence of ESI within Mexico’s *Salmonella* Infantis population. Considering the isolates sequenced in the last 17 years, ESI variants account for >60% of serovar Infantis isolates across sources and geographical regions. Our results suggest that this overrepresentation occurred recently, leading to the emergence of this lineage. Although ESI circulation was mainly observed in chickens, it also occurred in surface waters, which appear to play a relevant role in the ecology and transmission of this pathogen. However, further research is required to better understand the epidemiology of ESI.

Our results also showed that local ESI variants share the typical increased virulence and strong MDR genotypes observed in clones circulating globally. Furthermore, most of the ESI strains identified in this study were collected from retail chicken meat, highlighting the risk of human exposure to these potentially deadly strains, as documented elsewhere [3,5, 25]. The magnitude of this risk is difficult to determine given the limited scope of this study. Hence, further research in this area is critically important, considering the rapid expansion of ESI in poultry and the potential transmission of pESI-like plasmids to other *Salmonella* serovars.

## Supplementary material

10.1099/mgen.0.001645 Uncited Supplementary Material 1.

10.1099/mgen.0.001645 Uncited Supplementary Material 2.

## Abbreviations

AMR: antimicrobial resistance
ESI: emergent *Salmonella* Infantis
LMIC: low- and middle-income countries
MDR: multidrug-resistant
ML: maximum-likelihood
NCBI: National Center for Biotechnology Information

## References

[1] Delgado-Suarez EJ, Puente-Cruz AL, Sánchez-Zamorano LM, Rubio-Lozano MS, Ballesteros-Nova NE (2026). Emergence of multidrug-resistant blaCTX-M-65/gyrA_D87Y clones among the circulating Salmonella Infantis population in Mexico. Figshare.

[2] World Health Organization (2024). WHO bacterial priority pathogens list, 2024: Bacterial pathogens of public health importance to guide research, development and strategies to prevent and control antimicrobial resistance. https://www.who.int/publications/i/item/9789240093461.

[3] Alvarez DM, Barrón-Montenegro R, Conejeros J, Rivera D, Undurraga EA (2023). A review of the global emergence of multidrug-resistant *Salmonella enterica* subsp. enterica serovar Infantis. Int J Food Microbiol.

[4] García-Soto S, Abdel-Glil MY, Tomaso H, Linde J, Methner U (2020). Emergence of multidrug-resistant *Salmonella enterica* Subspecies *enterica* serovar Infantis of multilocus sequence type 2283 in German broiler farms. Front Microbiol.

[5] Tyson GH, Li C, Harrison LB, Martin G, Hsu C-H (2021). A multidrug-resistant *Salmonella* Infantis clone is spreading and recombining in the United States. Microb Drug Resist.

[6] Mughini-Gras L, van Hoek AHAM, Cuperus T, Dam-Deisz C, van Overbeek W (2021). Prevalence, risk factors and genetic traits of *Salmonella* Infantis in Dutch broiler flocks. Vet Microbiol.

[7] Regalado-Pineda ID, Rodarte-Medina R, Resendiz-Nava CN, Saenz-Garcia CE, Castañeda-Serrano P (2020). Three-Year longitudinal study: prevalence of *Salmonella Enterica* in chicken meat is higher in supermarkets than wet markets from Mexico. Foods.

[8] Godínez-Oviedo A, Tamplin ML, Bowman JP, Hernández-Iturriaga M (2020). *Salmonella enterica* in Mexico 2000-2017: epidemiology, antimicrobial resistance, and prevalence in food. Foodborne Pathog Dis.

[9] National center for biotechnology information (2025). NCBI Pathogen Detection. https://www.ncbi.nlm.nih.gov/pathogens/.

[10] Godínez-Oviedo A, Cabrera-Díaz E, Palacios-Marmolejo A, Pérez-Covarrubias OB, Vargas-Daniel RC (2022). Detection, quantification, and characterization of *Salmonella enterica* in mango, tomato, and raw chicken purchased in the central region of Mexico. J Food Sci.

[11] Gómez-Baltazar A, Godínez-Oviedo A, Vázquez-Marrufo G, Vázquez-Garcidueñas MS, Hernández-Iturriaga M (2023). Genomic analysis of the MLST population structure and antimicrobial resistance genes associated with *Salmonella enterica* in Mexico. Genome.

[12] Gómez-Baltazar A, Godínez-Oviedo A, Segura-García LE, Hernández-Pérez CF, Hernández-Iturriaga M (2024). Genomic diversity of *Salmonella enterica* isolated from raw chicken at retail establishments in Mexico. Int J Food Microbiol.

[13] Bankevich A, Nurk S, Antipov D, Gurevich AA, Dvorkin M (2012). SPAdes: a new genome assembly algorithm and its applications to single-cell sequencing. J Comput Biol.

[14] Yoshida CE, Kruczkiewicz P, Laing CR, Lingohr EJ, Gannon VPJ (2016). The *Salmonella* In Silico Typing Resource (SISTR): An Open Web-Accessible Tool for Rapidly Typing and Subtyping Draft Salmonella Genome Assemblies. PLoS One.

[15] Aviv G, Tsyba K, Steck N, Salmon-Divon M, Cornelius A (2014). A unique megaplasmid contributes to stress tolerance and pathogenicity of an emergent *Salmonella enterica* serovar Infantis strain. Environ Microbiol.

[16] Grant JR, Enns E, Marinier E, Mandal A, Herman EK (2023). Proksee: in-depth characterization and visualization of bacterial genomes. Nucleic Acids Res.

[17] Kaas RS, Leekitcharoenphon P, Aarestrup FM, Lund O (2014). Solving the problem of comparing whole bacterial genomes across different sequencing platforms. PLoS One.

[18] Minh BQ, Schmidt HA, Chernomor O, Schrempf D, Woodhams MD (2020). IQ-TREE 2: new models and efficient methods for phylogenetic inference in the genomic era. Mol Biol Evol.

[19] Naser-Khdour S, Minh BQ, Zhang W, Stone EA, Lanfear R (2019). The prevalence and impact of model violations in phylogenetic analysis. Genome Biol Evol.

[20] Letunic I, Bork P (2024). Interactive Tree of Life (iTOL) v6: recent updates to the phylogenetic tree display and annotation tool. Nucleic Acids Res.

[21] Rambaut A, Lam TT, Max Carvalho L, Pybus OG (2016). Exploring the temporal structure of heterochronous sequences using TempEst (formerly Path-O-Gen). Virus Evol.

[22] de Bernardi Schneider A, Ford CT, Hostager R, Williams J, Cioce M (2020). StrainHub: a phylogenetic tool to construct pathogen transmission networks. Bioinformatics.

[23] (2021). Mesquite: A modular system for evolutionary analysis. http://www.mesquiteproject.org.

[24] Alba P, Leekitcharoenphon P, Carfora V, Amoruso R, Cordaro G (2020). Molecular epidemiology of *Salmonella* Infantis in Europe: insights into the success of the bacterial host and its parasitic pESI-like megaplasmid. Microb Genom.

[25] Mattock J, Chattaway MA, Hartman H, Dallman TJ, Smith AM (2024). A one health perspective on *Salmonella enterica* serovar Infantis, an emerging human multidrug-resistant pathogen. *Emerg Infect Dis*.

[26] Mejía L, Medina JL, Bayas R, Salazar CS, Villavicencio F (2020). Genomic epidemiology of *Salmonella* Infantis in ecuador: from poultry farms to human infections. Front Vet Sci.

[27] Ballesteros-Nova NE, Sánchez S, Steffani JL, Sierra LC, Chen Z (2022). Genomic epidemiology of *Salmonella enterica* circulating in surface waters used in agriculture and aquaculture in central Mexico. Appl Environ Microbiol.

[28] Rocha AD de L, Ferrari RG, Pereira WE, de Lima LA, Givisiez PEN (2022). Revisiting the biological behavior of *Salmonella enterica* in hydric resources: a meta-analysis study addressing the critical role of environmental water on food safety and public health. Front Microbiol.

[29] Gymoese P, Kiil K, Torpdahl M, Østerlund MT, Sørensen G (2019). WGS based study of the population structure of *Salmonella enterica* serovar Infantis. BMC Genomics.

[30] Aviv G, Rahav G, Gal-Mor O (2016). Horizontal transfer of the *Salmonella enterica* serovar Infantis resistance and virulence plasmid pESI to the gut microbiota of warm-blooded hosts. mBio.

