# Supplementary material for: Emergence of multidrug-resistant blaCTX-M-65/gyrA_D87Y clones among the circulating Salmonella Infantis population in Mexico
Source: Microb Genom. 2026 Feb 16;12(2):001645. doi: 10.1099/mgen.0.001645 (PMC12908943; doi:10.1099/mgen.0.001645)
Supplement: Uncited Supplementary Material 1. [file mgen-12-01645-s001.pdf]

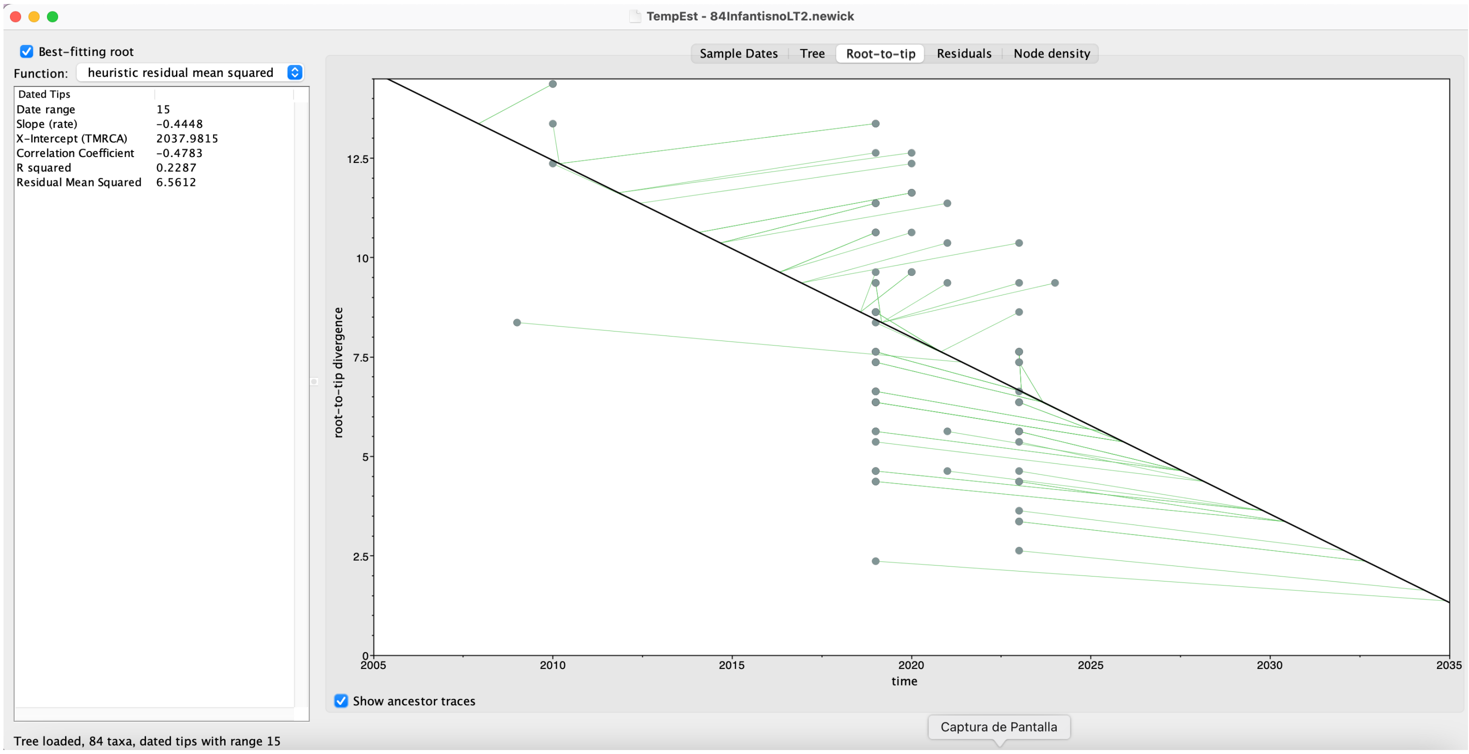

Fig. S1. Panel of the TempEst regression model between the genetic divergence through time and sampling dates showing the model statistics (left) and the root-to-tip regression line (right). Notice the negative slope and the variability in both axes in relation to the predicted values for most isolates on the regression line. Model created with the same dataset used for generating the ML phylogenetic tree used for the reconstruction of ancestral isolation sources (Fig. 5).

Fig. S2. BLAST atlas of emergent *Salmonella* Infantis plasmid (pESI) among 191 *Salmonella* Infantis strains. The gray line corresponds to the backbone and the blue arrows below correspond to pESI's reference coding sequences. Above the gray line, each slot reports the regions with shared synteny between genomes and the reference sequence, whereas blank spaces indicate a lack of synteny. The NCBI accession of strains are reported on the right side of the atlas, with those highlighted in red font corresponding to strains lacking pESI.

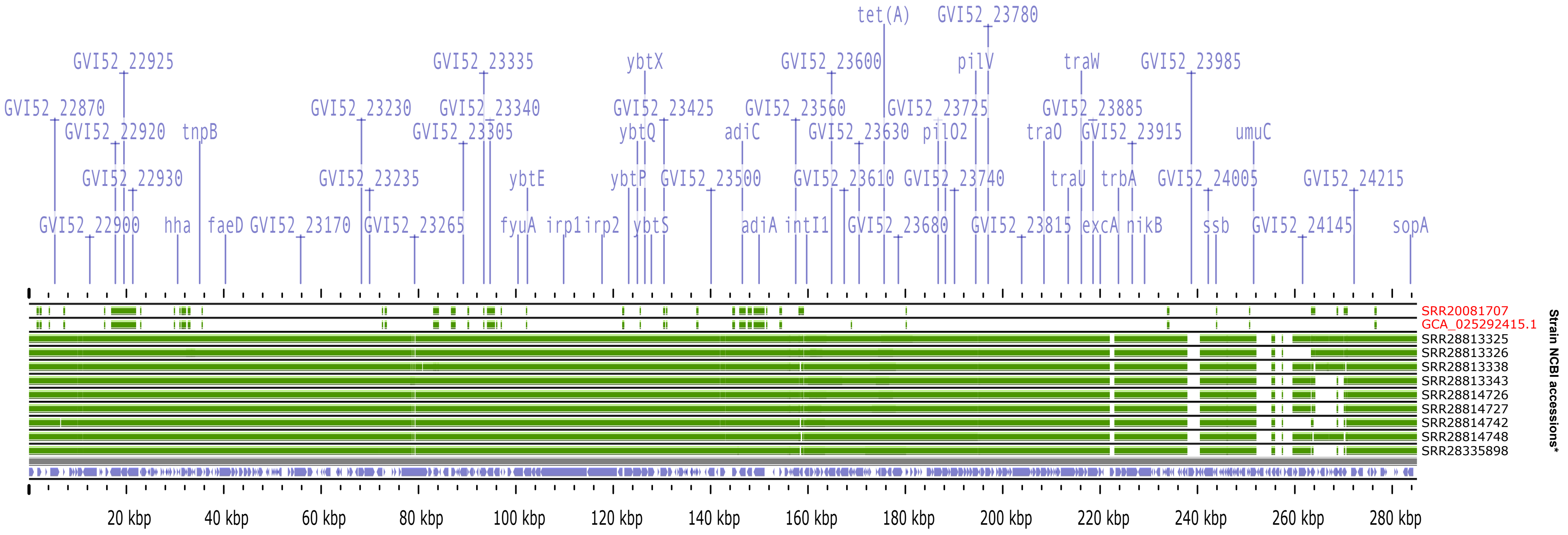

pESI (reference sequence CP047882)

*Strains lacking pESI-like plasmids are indicated in red font

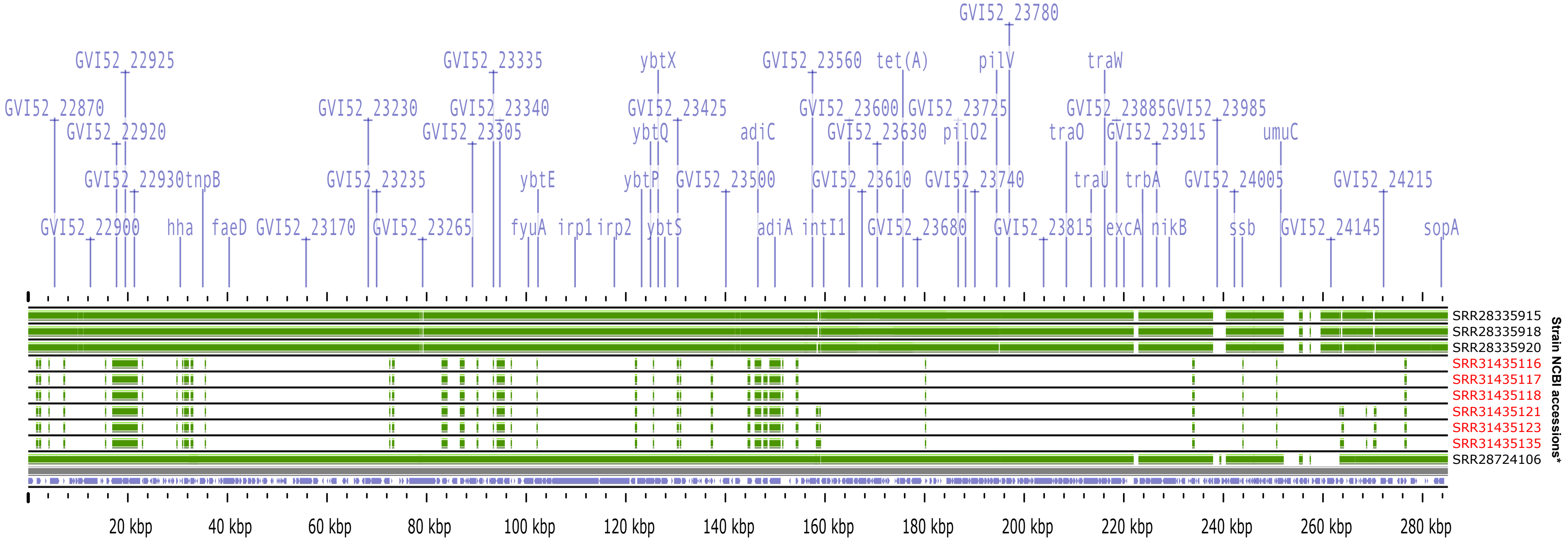

pESI (reference sequence CP047882)

*Strains lacking pESI-like plasmids are indicated in red font

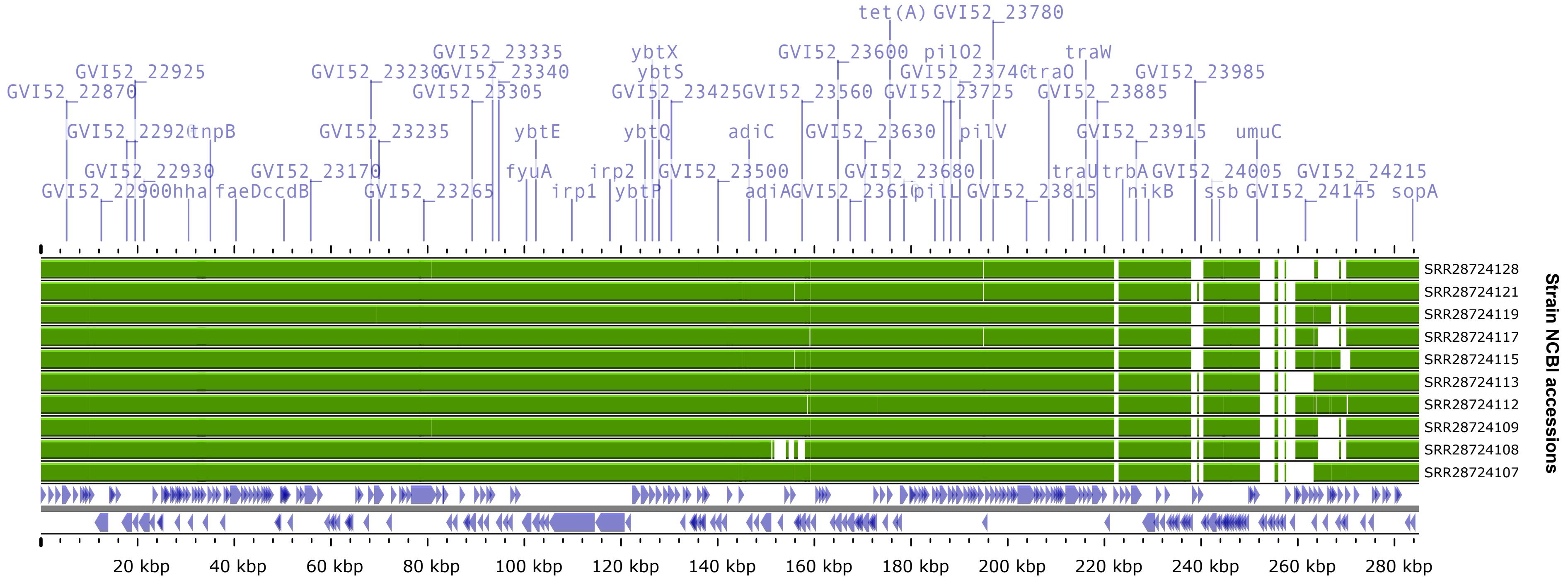

pESI (reference sequence CP047882)

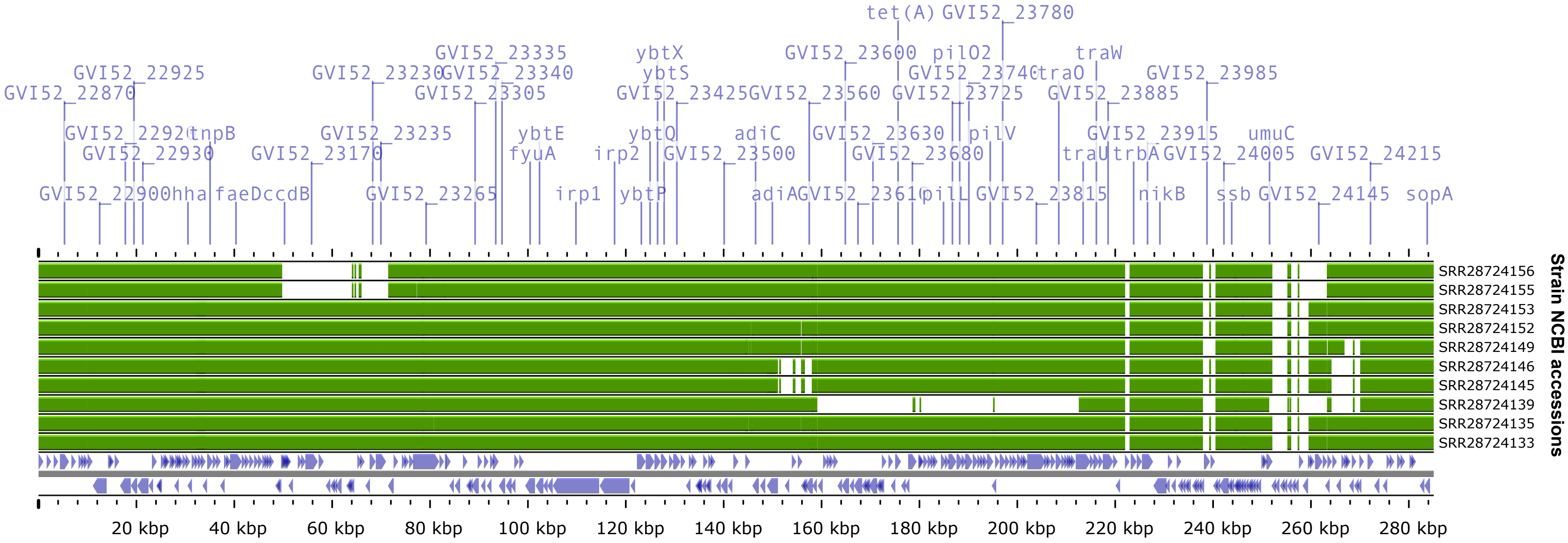

pESI (reference sequence CP047882)

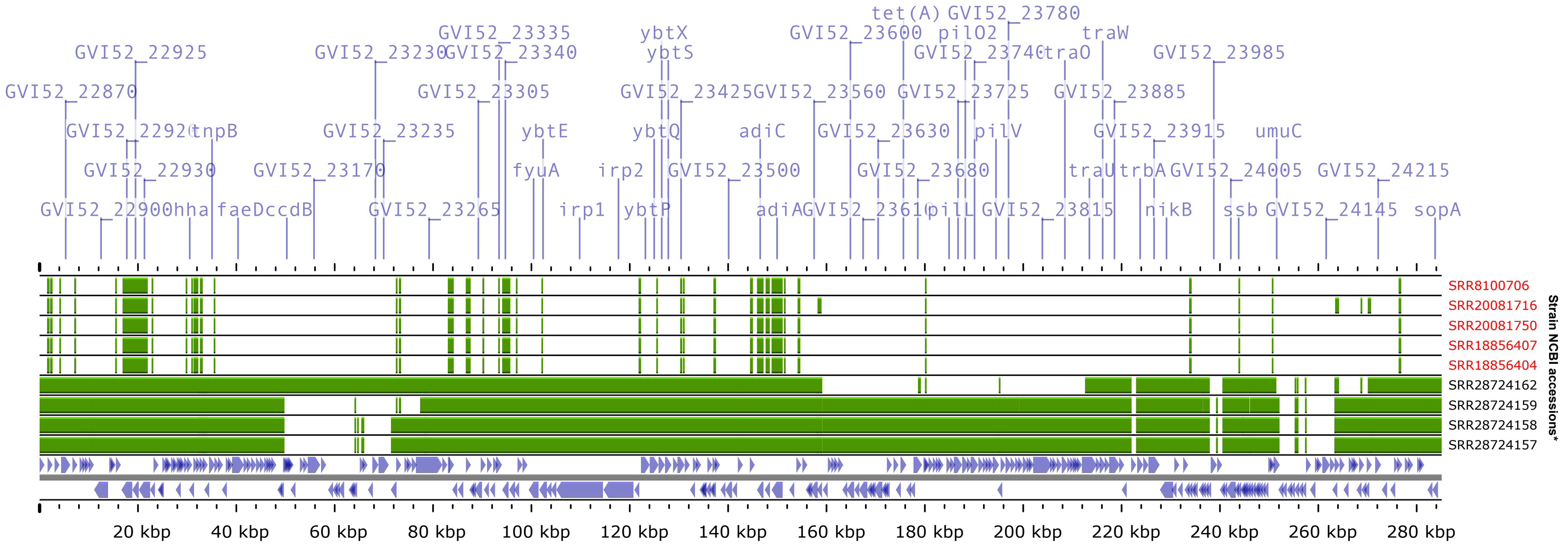

pESI (reference sequence CP047882)

*Strains lacking pESI-like plasmids are indicated in red font

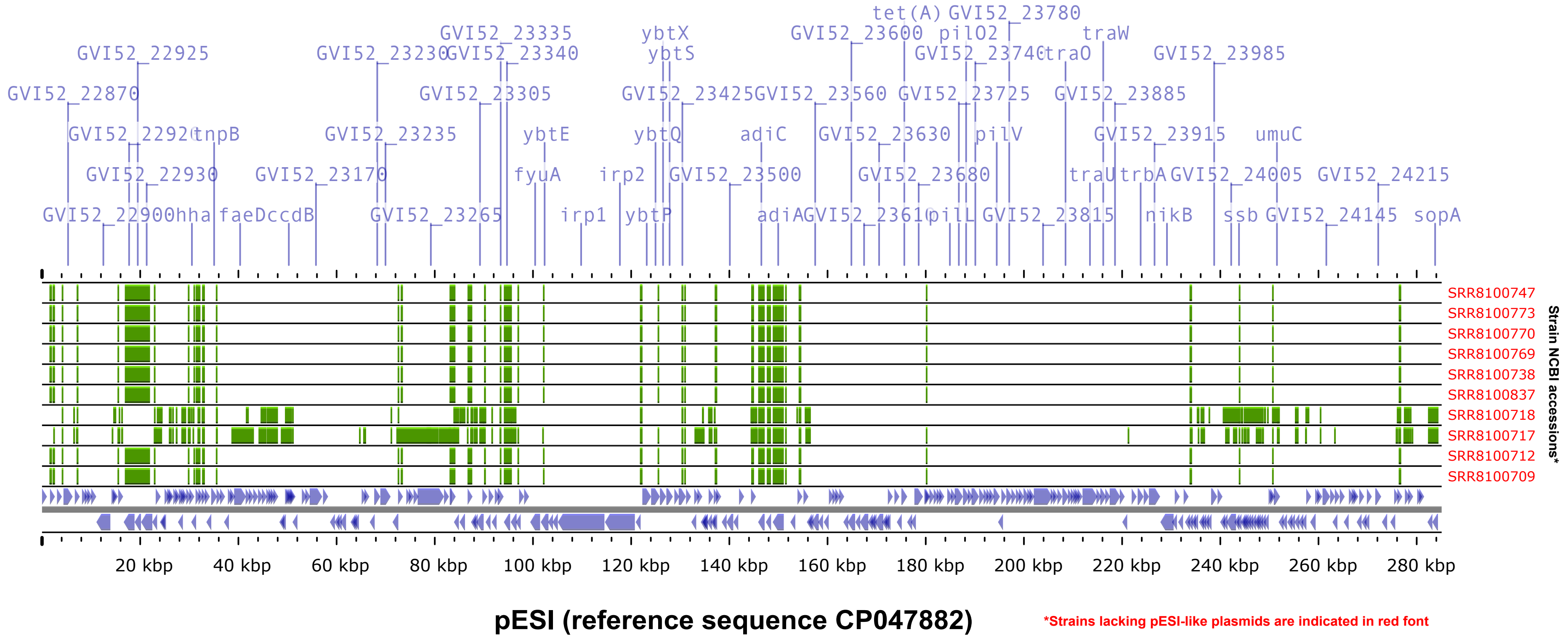
tet(A) GVI52_23780
GVI52_23335
ybtX
GVI52_23600
pilO2
traW
GVI52_22925
GVI52_23230
GVI52_23340
ybtS
GVI52_23740
traO
GVI52_23985
GVI52_22870
GVI52_23305
GVI52_23425
GVI52_23560
GVI52_23725
GVI52_23885
GVI52_22920
tnpB
GVI52_23235
ybtE
ybtQ
adiC
GVI52_23630
pilV
GVI52_23915
umuC
GVI52_22930
GVI52_23170
fyuA
irp2
GVI52_23500
GVI52_23680
traU
trbA
GVI52_24005
GVI52_24215
GVI52_22900
hha
faeD
ccdB
GVI52_23265
irp1
ybtP
adiA
GVI52_23610
pilL
GVI52_23815
nikB
ssb
GVI52_24145
sopA
SRR8100747
SRR8100773
SRR8100770
SRR8100769
SRR8100738
SRR8100837
SRR8100718
SRR8100717
SRR8100712
SRR8100709
Strain NCBI accessions*
20 kbp
40 kbp
60 kbp
80 kbp
100 kbp
120 kbp
140 kbp
160 kbp
180 kbp
200 kbp
220 kbp
240 kbp
260 kbp
280 kbp
pESI (reference sequence CP047882)
*Strains lacking pESI-like plasmids are indicated in red font

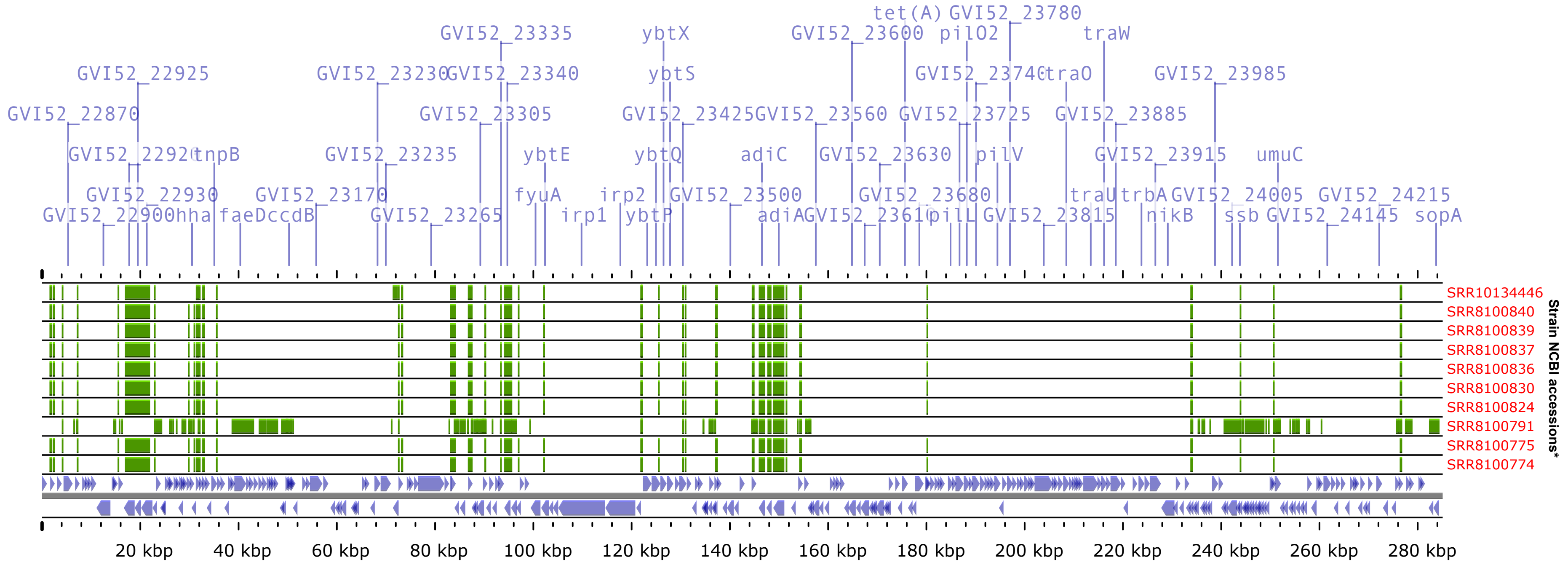

pESI (reference sequence CP047882)

*Strains lacking pESI-like plasmids are indicated in red font

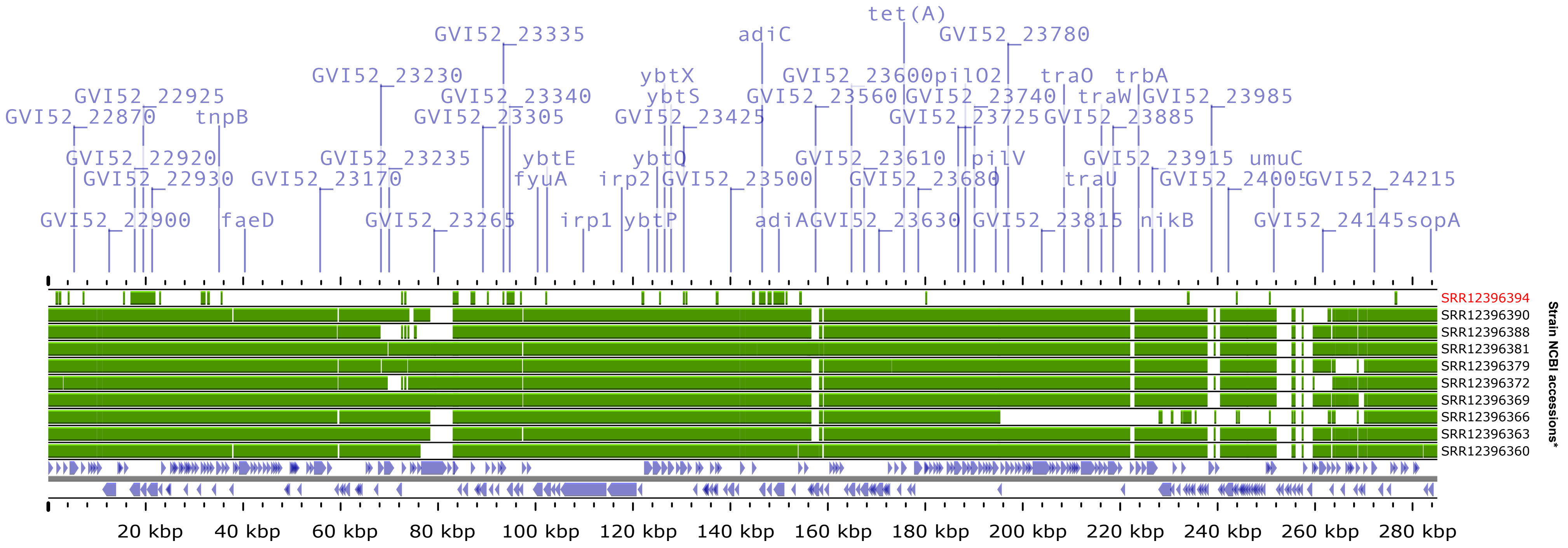

pESI (reference sequence CP047882)

*Strains lacking pESI-like plasmids are indicated in red font

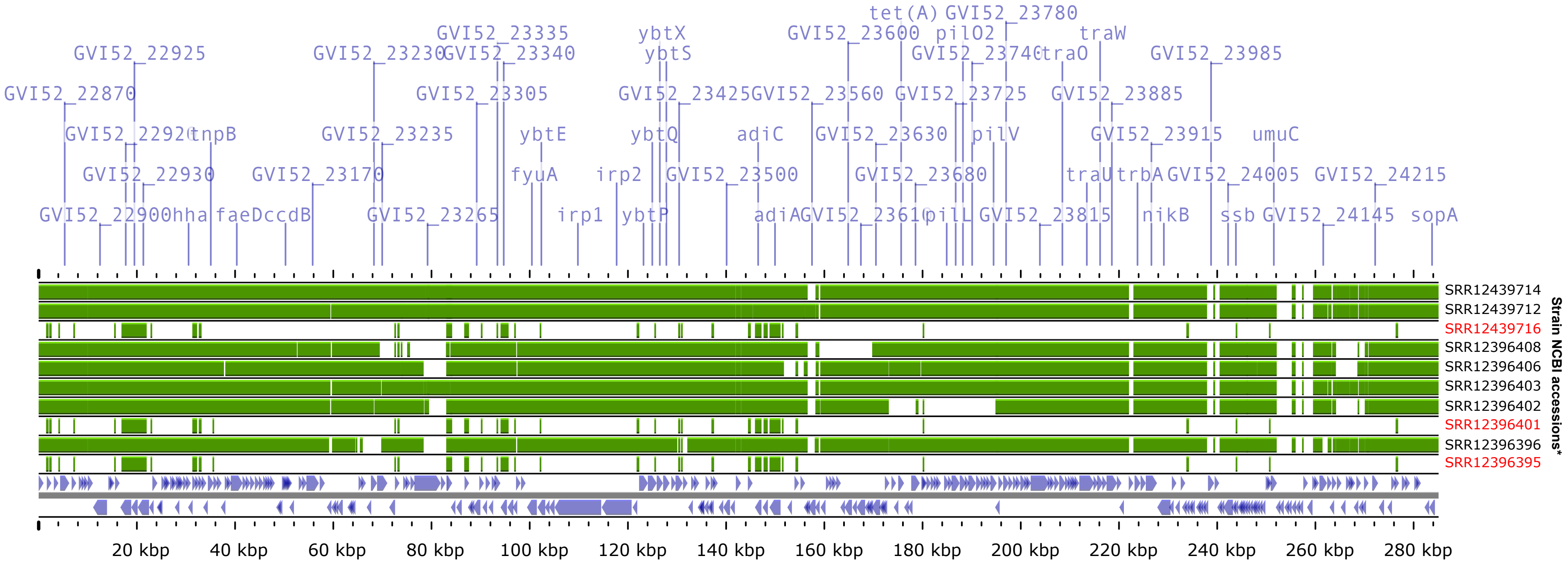

pESI (reference sequence CP047882)

*Strains lacking pESI-like plasmids are indicated in red font

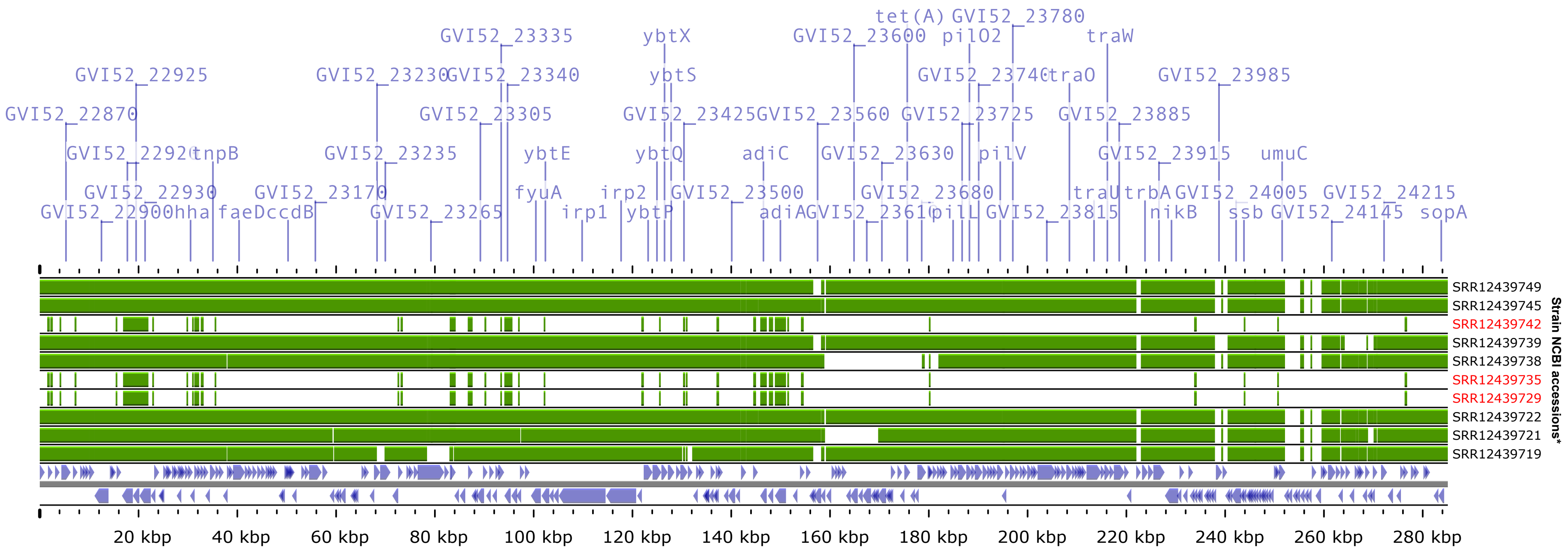

pESI (reference sequence CP047882)

*Strains lacking pESI-like plasmids are indicated in red font

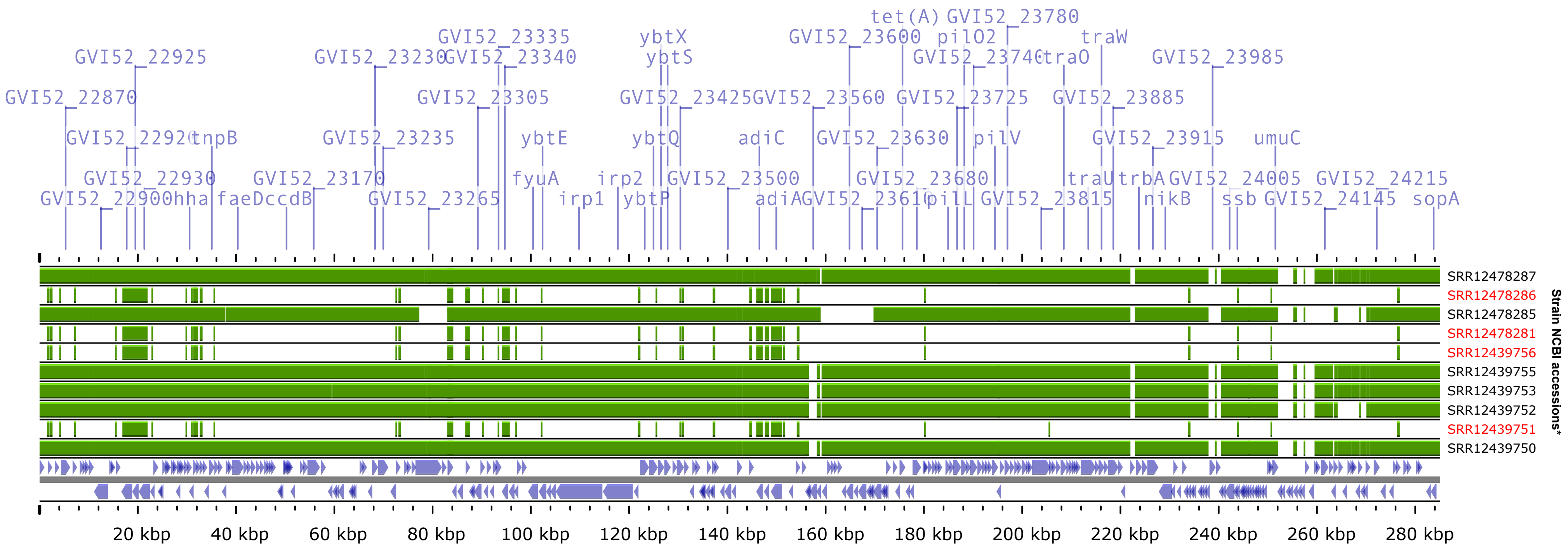

pESI (reference sequence CP047882)

*Strains lacking pESI-like plasmids are indicated in red font

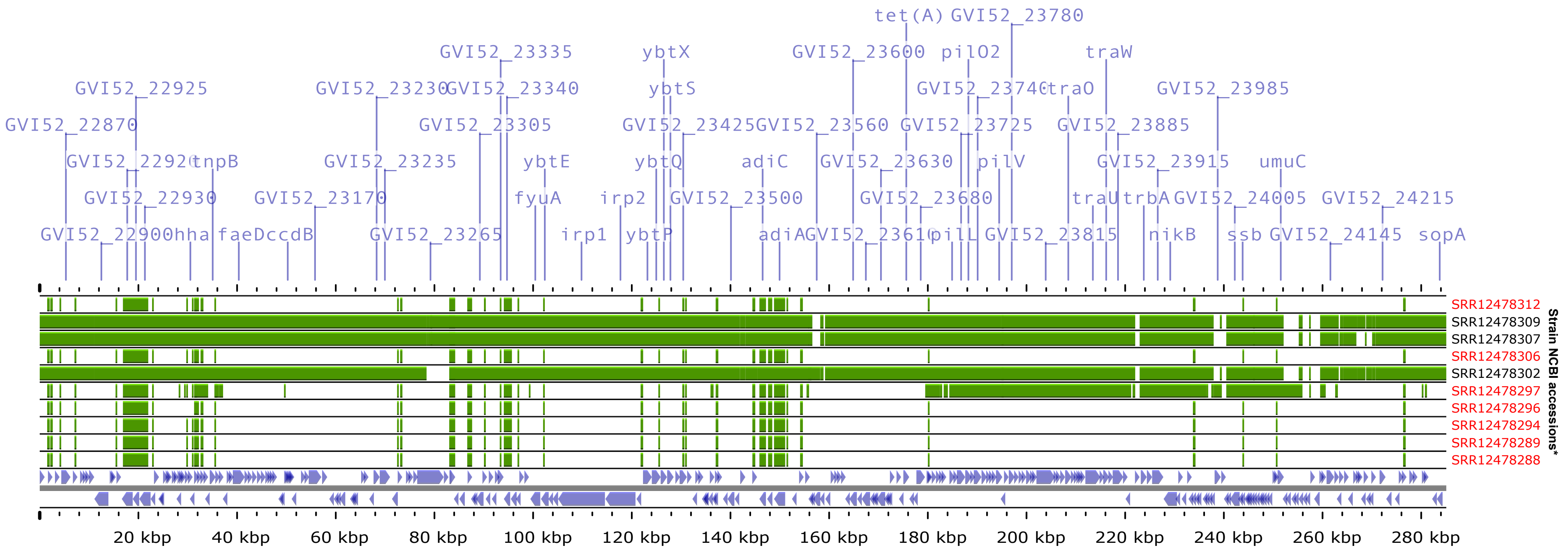

pESI (reference sequence CP047882)

*Strains lacking pESI-like plasmids are indicated in red font

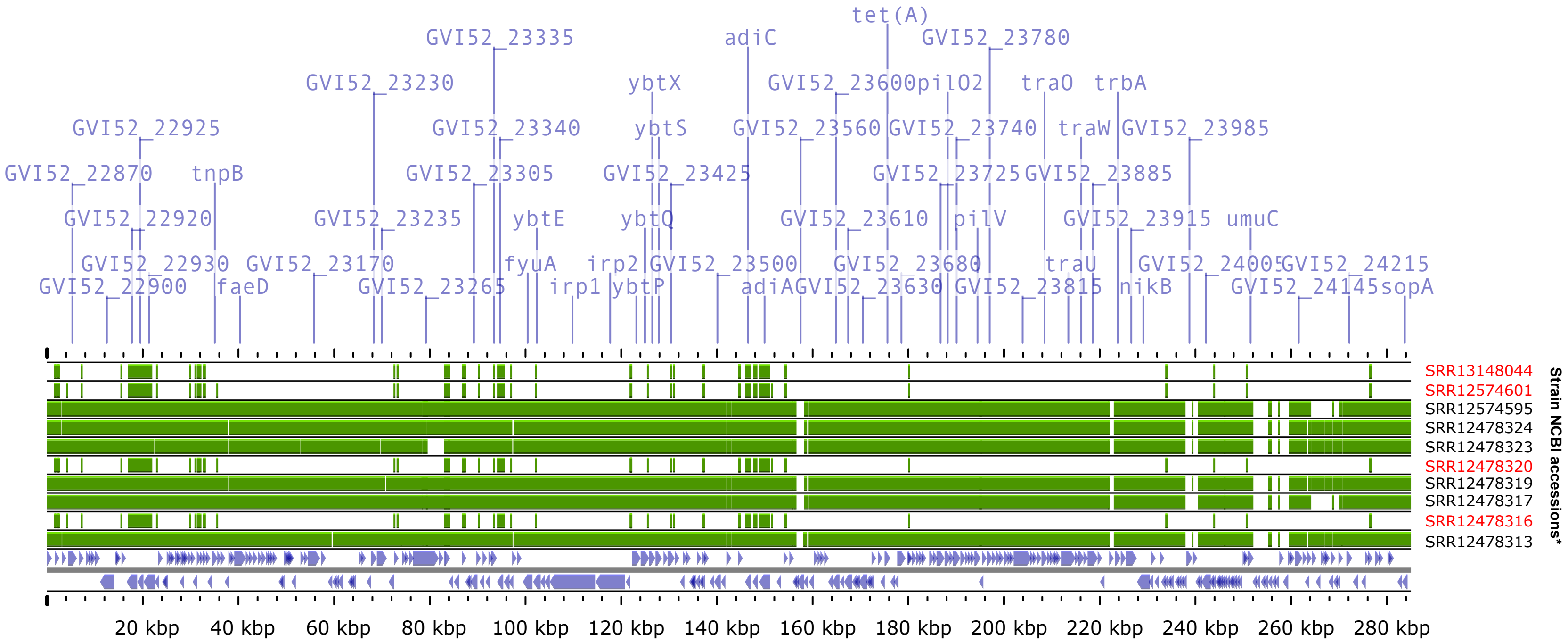

pESI (reference sequence CP047882)

*Strains lacking pESI-like plasmids are indicated in red font

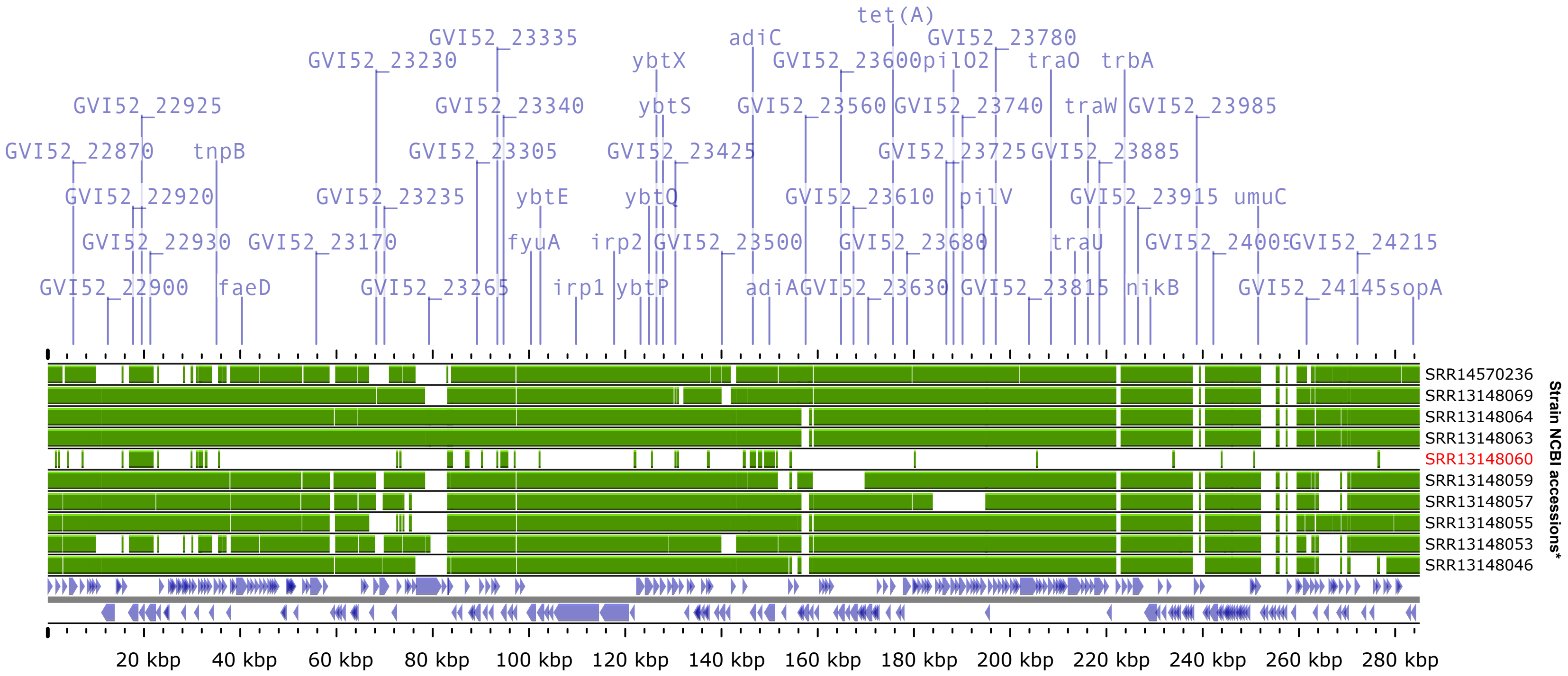

pESI (reference sequence CP047882)

*Strains lacking pESI-like plasmids are indicated in red font

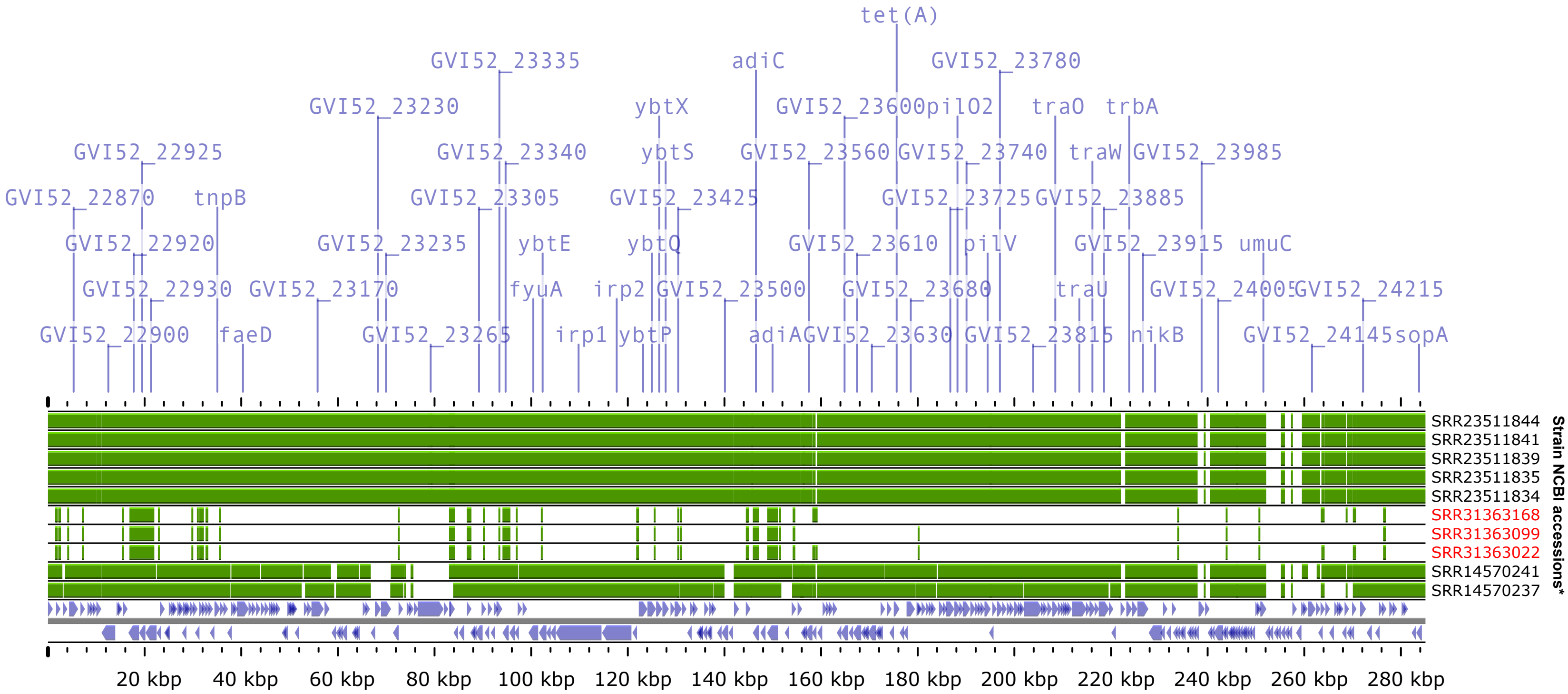

pESI (reference sequence CP047882)

*Strains lacking pESI-like plasmids are indicated in red font

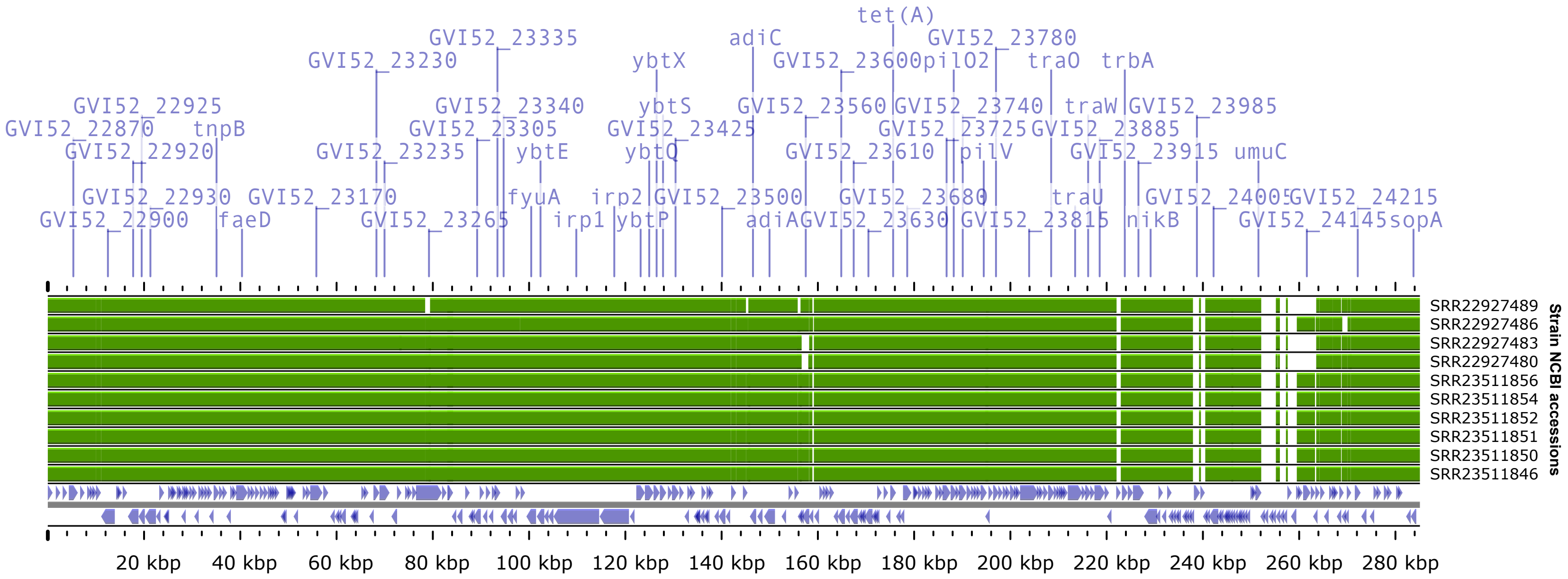

pESI (reference sequence CP047882)

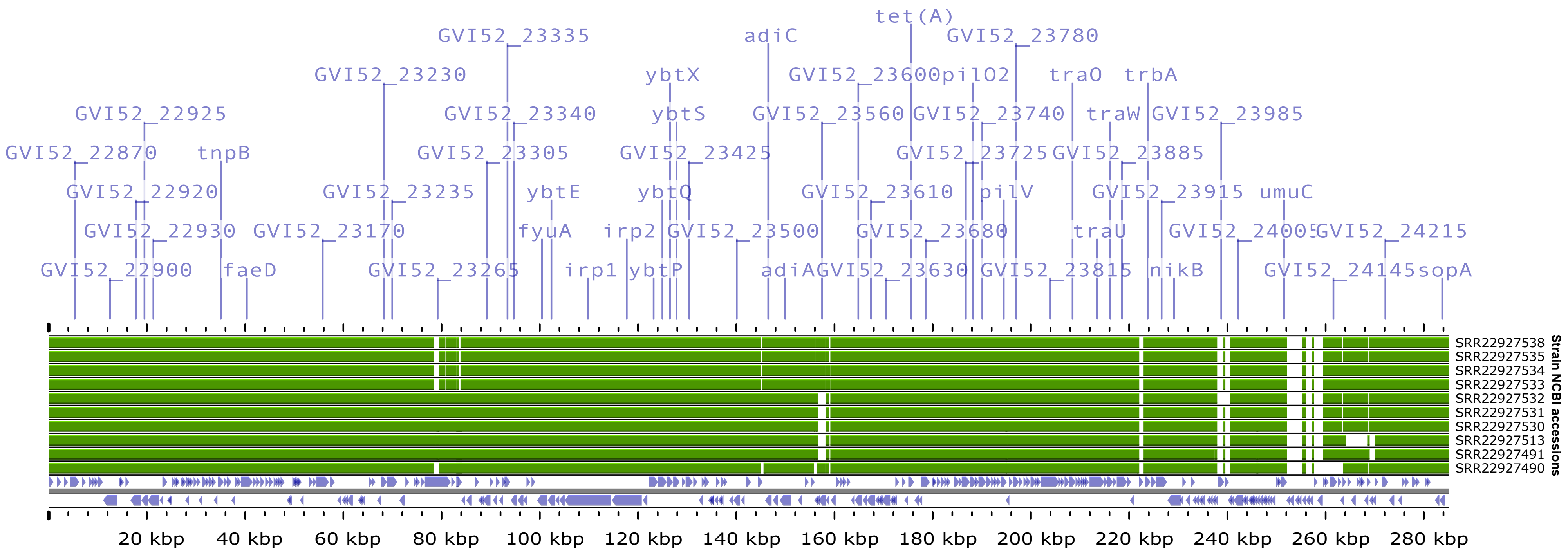

pESI (reference sequence CP047882)

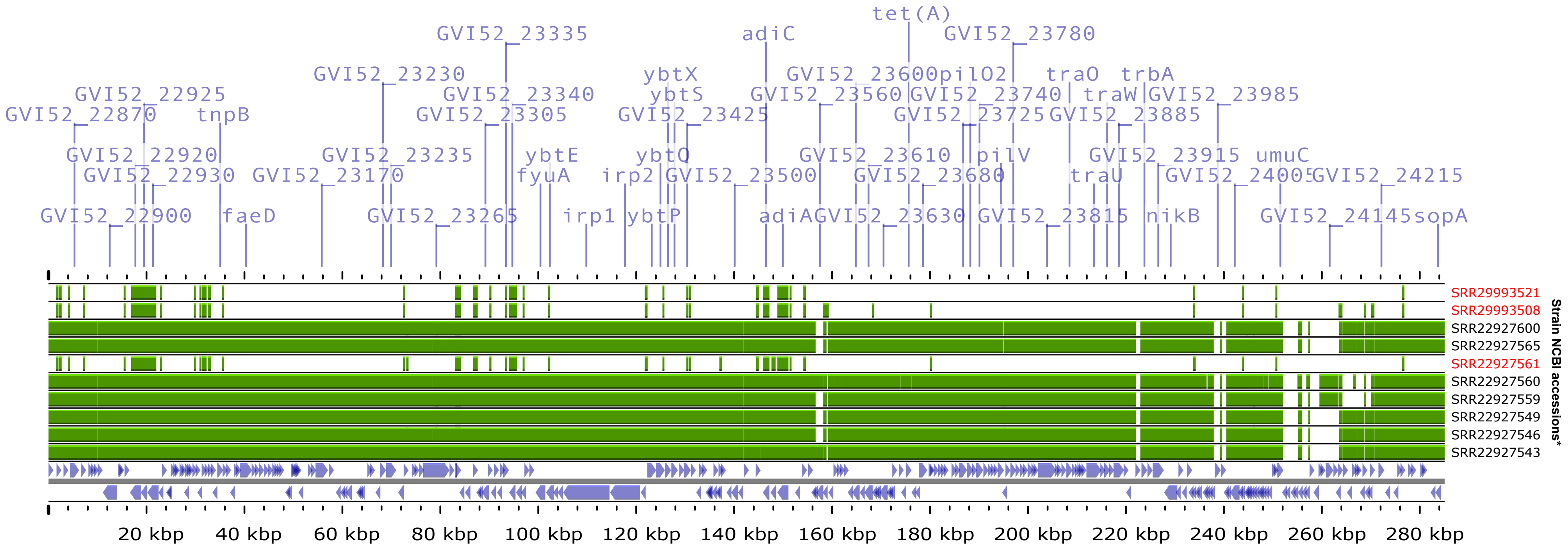

pESI (reference sequence CP047882)

*Strains lacking pESI-like plasmids are indicated in red font

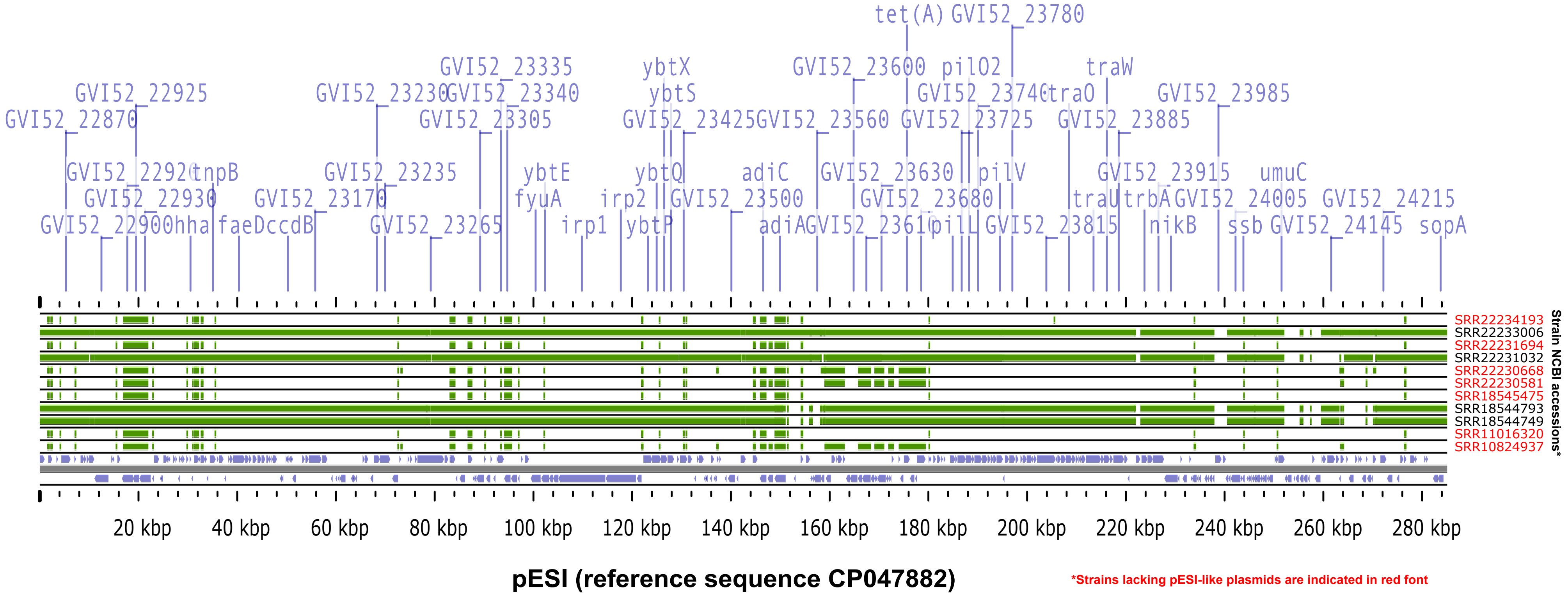
tet(A) GVI52_23780
GVI52_23335 ybtX GVI52_23600 pilO2 traW
GVI52_22925 GVI52_23230 GVI52_23340 ybtS GVI52_23740 traO GVI52_23985
GVI52_22870 GVI52_23305 GVI52_23425 GVI52_23560 GVI52_23725 GVI52_23885
GVI52_22920 tnpB GVI52_23235 ybtE ybtQ adiC GVI52_23630 pilV GVI52_23915 umuC
GVI52_22930 GVI52_23170 fyuA irp2 GVI52_23500 GVI52_23680 traU trbA GVI52_24005 GVI52_24215
GVI52_22900 hha faeD ccdB GVI52_23265 irp1 ybtP adiA GVI52_23610 pilL GVI52_23815 nikB ssb GVI52_24145 sopA
SRR22234193
SRR22233006
SRR22231694
SRR22231032
SRR22230668
SRR22230581
SRR18545475
SRR18544793
SRR18544749
SRR11016320
SRR10824937
Strain NCBI accessions*
20 kbp
40 kbp
60 kbp
80 kbp
100 kbp
120 kbp
140 kbp
160 kbp
180 kbp
200 kbp
220 kbp
240 kbp
260 kbp
280 kbp
pESI (reference sequence CP047882)
*Strains lacking pESI-like plasmids are indicated in red font

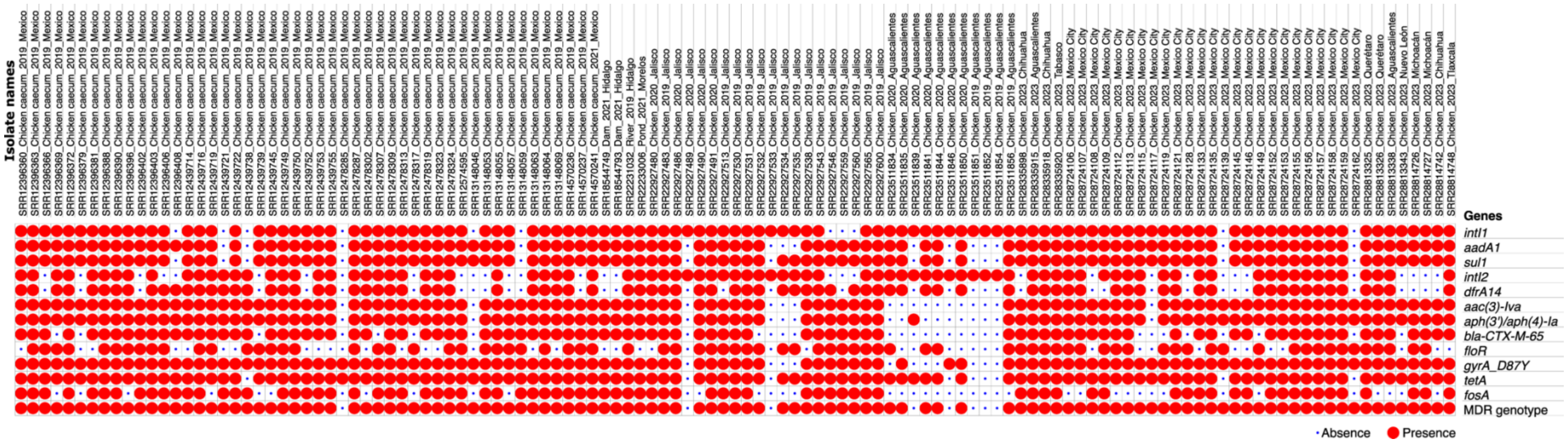

Fig. S3. Heatmap showing the occurrence of class-1/class-2 integrons, AMR genes, and multidrug (MDR) genotypes across 121 *Salmonella* Infantis isolates carrying pESI-like plasmids.

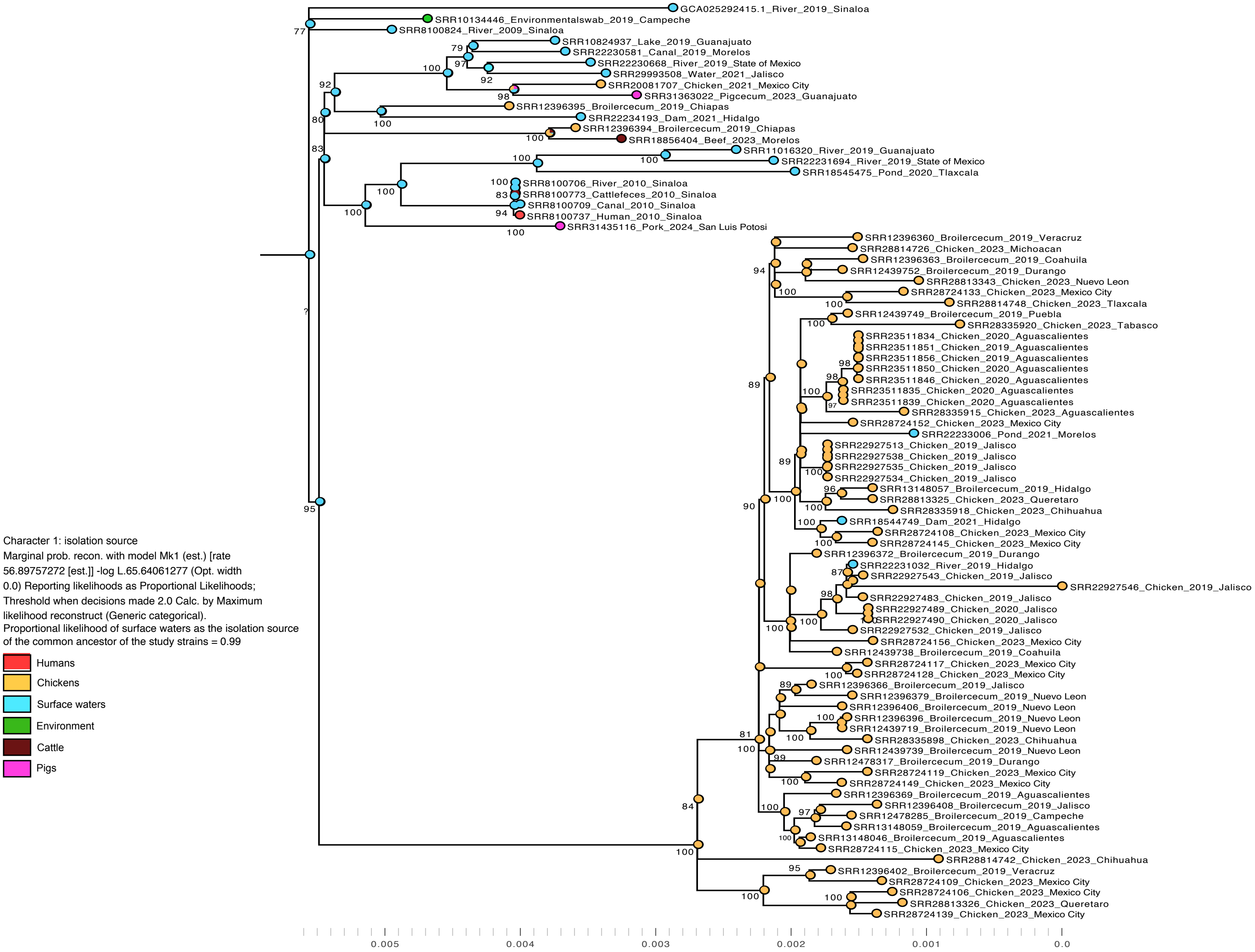

Fig. S4. Isolation source history at ancestral nodes in a phylogenetic tree of 84 Salmonella Infantis isolates by the maximum likelihood method. The reconstructed isolation source is color-coded at each ancestral node, with the corresponding estimated proportional likelihood. Statistical support (percent bootstrap) is indicated on the branches. NCBI accessions, source, year of collection, and location are indicated on the tip labels. ESI strains are indicated using bold font.
